# A microfluidic platform for the controlled synthesis of architecturally complex liquid crystalline nanoparticles

**DOI:** 10.1038/s41598-023-39205-3

**Published:** 2023-08-04

**Authors:** Colin P. Pilkington, Claudia Contini, Joseph D. Barritt, Paul A. Simpson, John M. Seddon, Yuval Elani

**Affiliations:** 1 Department of Chemistry, Molecular Science Research Hub, Imperial College London, 82 Wood Lane, London, W12 0BZ UK; 2 Department of Chemical Engineering, Imperial College London, Exhibition Road, London, SW7 2AZ UK; 3 Department of Life Sciences, Imperial College London, Exhibition Road, London, SW7 2AZ UK; 4 Department of Life Sciences, Centre for Structural Biology, Imperial College London, Exhibition Road, London, SW7 2AZ UK

**Keywords:** Biophysics, Membrane biophysics, Drug delivery, Nanoscale materials

## Abstract

Soft-matter nanoparticles are of great interest for their applications in biotechnology, therapeutic delivery, and in vivo imaging. Underpinning this is their biocompatibility, potential for selective targeting, attractive pharmacokinetic properties, and amenability to downstream functionalisation. Morphological diversity inherent to soft-matter particles can give rise to enhanced functionality. However, this diversity remains untapped in clinical and industrial settings, and only the simplest of particle architectures [spherical lipid vesicles and lipid/polymer nanoparticles (LNPs)] have been routinely exploited. This is partially due to a lack of appropriate methods for their synthesis. To address this, we have designed a scalable microfluidic hydrodynamic focusing (MHF) technology for the controllable, rapid, and continuous production of lyotropic liquid crystalline (LLC) nanoparticles (both cubosomes and hexosomes), colloidal dispersions of higher-order lipid assemblies with intricate internal structures of 3-D and 2-D symmetry. These particles have been proposed as the next generation of soft-matter nano-carriers, with unique fusogenic and physical properties. Crucially, unlike alternative approaches, our microfluidic method gives control over LLC size, a feature we go on to exploit in a fusogenic study with model cell membranes, where a dependency of fusion on particle diameter is evident. We believe our platform has the potential to serve as a tool for future studies involving non-lamellar soft nanoparticles, and anticipate it allowing for the rapid prototyping of LLC particles of diverse functionality, paving the way toward their eventual wide uptake at an industrial level.

## Introduction

Advances in soft-matter nanotechnology, and the adaptation of biomimetic self-assembled nanoparticles for use in industrial, clinical and academic settings has called on researchers to re-evaluate generation methods^1–5^. This is especially important where greater compositional and/or morphological complexity is required^4^, in fields such as targeted drug delivery^6–8^, catalysis^9,10^, food science^11,12^ and vaccinology^4,13,14^. The successful integration of these nano-carriers into modern medicine has recently been demonstrated with vaccine efforts against COVID-19^15^. The internal structure, shape, and size of nano-carriers can have a profound effect on stability, encapsulation efficiency, fusogenic behaviour, cellular uptake, pharmacokinetics, and stimulus-response^12,14,16^ However, most research at a clinical and industrial level has focused primarily on simple spherical liposomes and solid lipid nanoparticles. To fully explore the functional potential granted by morphological variation it is thus imperative that a scalable, efficient, and easily controlled platform be established for the synthesis of nanostructures of enhanced architectural complexity.

Cubosomes and hexosomes are stable dispersions of lyotropic liquid crystalline mesophases (LLCs) and are prime examples of such higher-order structures, with unique physical properties closely related to their morphology^4,12,17,18^. Cubosomes, consisting of stabilised particles of lipid bilayers draped over minimal surfaces (primitive, diamond, gyroid), have recently been the focus of several important publications dealing with their potential as drug delivery vehicles^19–22^. Their highly curved internal structure has been shown to undergo fusion via lipid incorporation with model membranes, as well as facilitating improved loading of hydrophobic, lipophilic, and fragile biomolecular cargo such as mRNA^17,18^. Hexosomes are soft nanoparticles with internal structures comprising of hexagonally packed lipid tubules with 2D symmetry. They have also been proposed as drug delivery vehicles capable of sustained release, their tightly wound tubular structures acting as barriers through which cargo must pass^18^.

Existing methods for LLC fabrication consist of high-energy shearing techniques like probe sonication and high-shear mixing^12,17,23,24^. Arduous optimisation steps are required in both, offering very little control over nanoparticle size, known to be a crucial variable for most envisaged applications. While polydispersity indices (PDI) of generated particles below 0.2 can be achieved (a general requirement in industry), batch-to-batch variation is difficult to avoid. Sonication in particular has reduced scalability, associated health hazards, and is largely unsuitable for the simultaneous encapsulation of fragile biomolecules (including mRNA, DNA and proteins), sensitive to high temperatures, pressures, and mechanical forces^25,26^. Considering the wide-spread interest in biomolecular therapeutics, this is a major impediment.

Microfluidic methods offer an exciting alternative to bulk methods for the manufacture of LLCs, which could in principle allow vastly superior control over important parameters such as particle size and composition^2–6^. Such platforms are modular, easy to use, and highly amenable to industrial scale-up^27^. Promising research has already been established, though size control and morphological heterogeneity remain critical issues, with some techniques requiring an off-chip post-processing step at elevated temperatures^28,29^.

**Figure 1:**
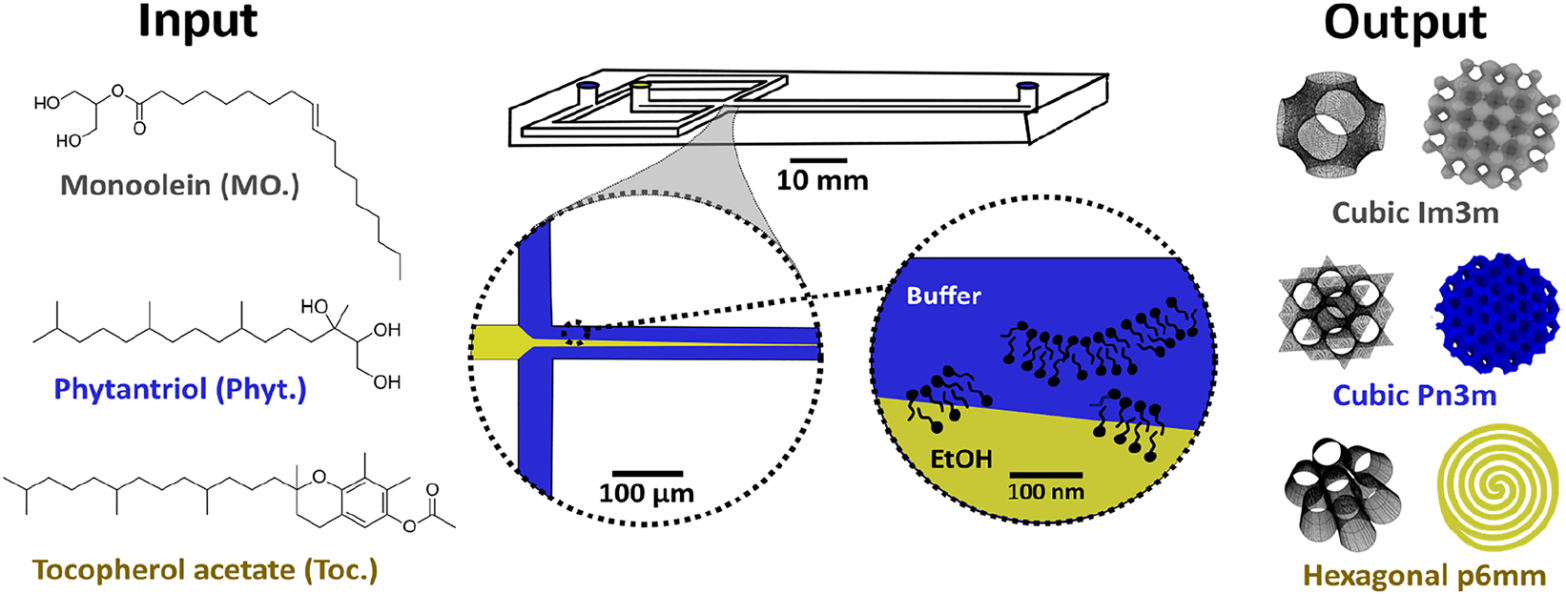
Graphical depiction of the microfluidic platform used to generate lyotropic liquid crystalline nanoparticles (LLCs). Syringes containing 1X PBS, Pluronic F-127 solution, and lipids dissolved in ethanol, were mounted on a syringe pump. The hydrodynamic flow regime was adjusted by varying the flow rate ratio between the central (lipid) and flanking (buffer) streams. Self-assembly occurs at the diffusion interface between the two streams. Further details of the microfluidic set-up can be found in the supplementary information Fig. S.3.

With this in mind, we present a microfluidic device for the continuous production of lyotropic liquid crystalline mesophase nanoparticles, exploiting the principles of microfluidic hydrodynamic focusing (MHF). Amphiphiles are dissolved in a water-miscible organic solvent that is hydrodynamically focused into a thin stream, along which a diffusion gradient is generated. Upon reaching solubility thresholds, amphiphiles self-assemble into micellar structures that aggregate to produce larger assemblies^30,31^. The nucleation-growth mechanism is controlled via flow rate ratios, and subsequently the hydrodynamic diameters of nanoparticles produced can be finely tuned^4,27,30,31^. Figure 1 illustrates the series of events in MHF-driven lipid assembly.

Cubosomes of both Pn3m and Im3m space group, and hexosomes (plane group p6mm) were generated by MHF. We show that by adjusting flow rates used, excellent size control at low dispersity values can be achieved, aligning with typical trends for liposomal MHF. Particles were stable at 37 °C, required no additional post-processing steps and were characterised using a combination of dynamic light scattering, small angle X-ray scattering, and cryo-transmission electron microscopy. To investigate the loading efficiency of each particle type, two spectroscopic assays were included, using hydrophilic and hydrophobic dyes. Finally, to demonstrate the value of size control, we present a simple fusogenic study using two LLC size populations. Smaller particles were seen to deliver a fluorescent payload, whereas larger particles did not. We expect this platform to present an efficient and modular route toward rapidly prototyping LLC particles of diverse composition and morphology, essential for future uptake at an industrial level.

## Results and discussion

### LLC generation via MHF

Cubosomes and hexosomes were prepared on-chip as described in the methods section. Briefly, a lipid of choice was dissolved in anhydrous ethanol and injected into the central channel via a syringe connected to a syringe pump. Aqueous buffer, containing the stabilising polymer Pluronic F-127, was injected into the flanking channels in the same way, and a hydrodynamic flow regime was generated. Lipids reached their respective solubility thresholds along the resulting diffusion gradient, aggregated into micellar structures, and eventually self-assembled into particles stabilised by F-127. The schematic in Fig. 1 depicts the process. Details of the set-up, concentrations used, flow rates and chip design can all be found in the supplementary information section (Fig. S.3).

### LLC nanoparticle characterisation

A multifaceted approach to particle characterisation was necessary here, combining several analytical techniques to best elucidate structural complexity. These techniques included dynamic light scattering (DLS), cryo-transmission electron microscopy with fast Fourier transform analysis (FFT), and small angle x-ray scattering (SAXS). The potential for microfluidic encapsulation of cargo into LLCs was also analysed using UV/vis, fluorescence spectroscopy, and confocal microscopy.

### Demonstrating size control

To assess the capability of this on-chip method to tune the size of generated nanoparticles, flow rate ratios (FRR) between the buffer (Q_buffer_) and ethanolic (Q_ethanol_) streams were varied from 5 to 60, with a total flow rate (TFR) of around 480 µL/min [see Eq. (1)].

At higher TFR values a broadening of size distributions was noted via DLS (polydispersity indices > 0.2) along with the co-existence of sub 60 nm particles (possibly liposomes), thought to arise from turbulence in the F-127/buffer stream with increased back pressure (see supplementary information Fig. S.3).

1 $$FRR= \frac{{Q}_{buffer}}{{Q}_{ethanol}}$$

**Figure 2:**
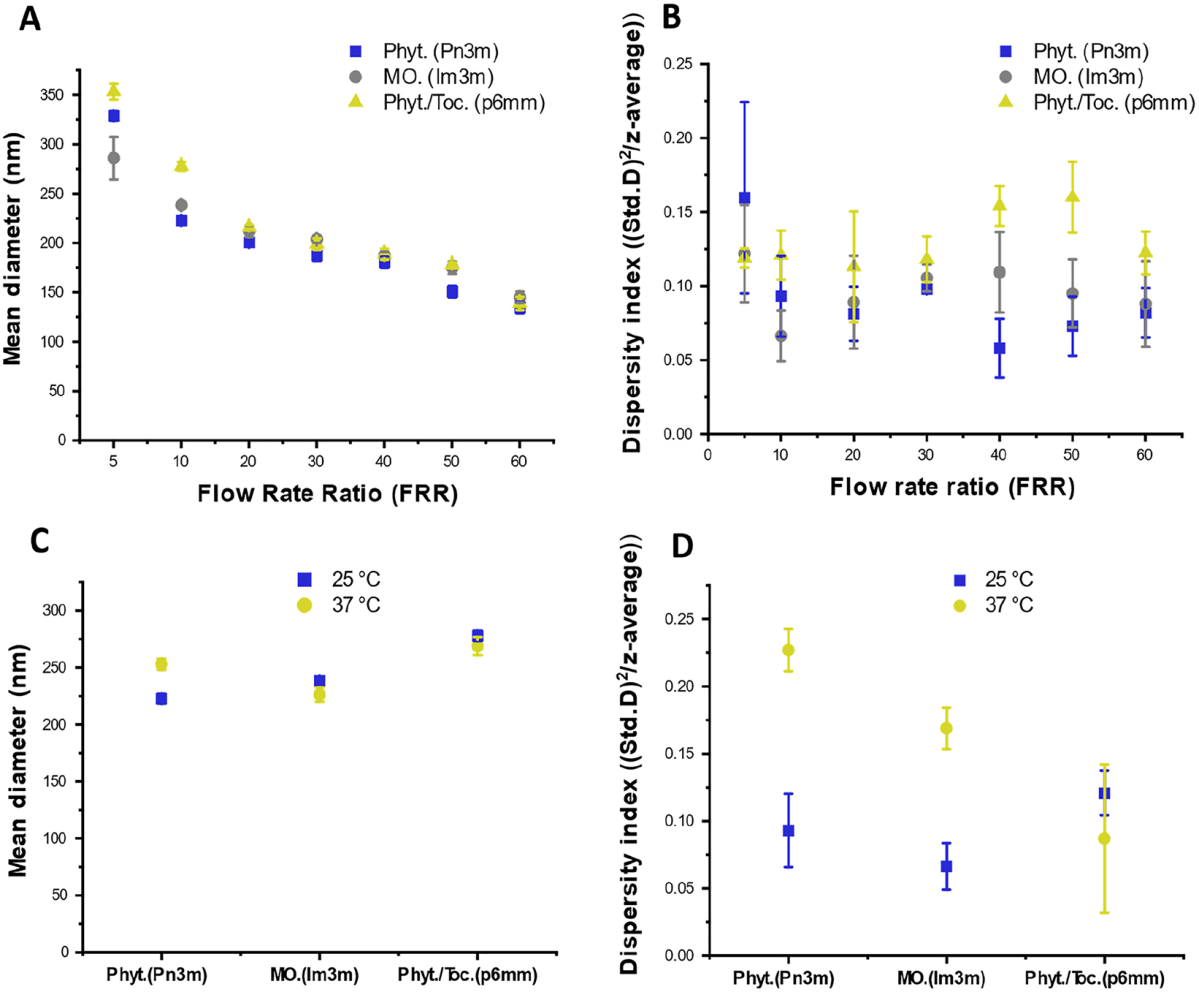
(**A**) Dynamic light scattering (DLS) at increasing flow rate ratios performed at room temperature, with (**B**) showing dispersity indices for the same data set. Error bars indicate standard deviation between mean diameter/dispersity index values collected from three separate experiments on three separate microfluidic chips. Size is varied from ~ 350 to ~ 130 nm by changing FRR values, thereby decreasing diffusion length and increasing overall number of nucleation points about which LLC nanoparticles are presumed to form. All DLS measurements were taken where correlogram intercepts were between 0.9 and 1 (indicating statistical reliability). Dispersity indices (PDI values) for each sample were below 0.2, related to the distribution range for each Gaussian plot, a general requirement for industry. No discernible trend was seen between these values and FRR. (**C)**. Mean diameter measurements for samples (generated with FRR 20) before and after incubation at 37 °C for 24 h, along with corresponding PDI values in (**D)** for the same data sets. It was noted that PDI error for hexosomes was larger after incubation, though values still fall within an acceptable range for PDI. We speculate that this may be to do with hexosomal particle growth being limited to a 2 directions (2-D symmetric). DLS methods assume a spherical hydrodynamic diameter, thus sizes may differ slightly between runs where particles are more faceted and/or rod-like. Error bars indicate standard deviations calculated as before. Negligible difference in hydrodynamic size was taken to infer minimal change in particle stability at elevated temperatures. See SI, Fig. S.2 for DLS details, including gaussian plots and correlograms.

Each FRR value was tested in triplicate using three separately prepared solutions (polymer and lipid) on three different PDMS chips to best account for any batch-to-batch variation. As shown in Fig. 2A, an inverse relationship between FRR and particle size (analysed by DLS) was seen for each composition used. Particles between around 130 nm and 300 nm were generated (dependant on FRR), with polydispersity indices (PDI) below 0.2 (shown in Fig. 2B). Many drug-delivery and nanoengineering applications require precise control over particle size distribution, rendering this a significant result. No change in size was observed between particles analysed immediately after collection and samples where residual ethanol was removed under a nitrogen stream, suggesting the presence of ethanol had a negligible effect on particle stability. A lower hydrodynamic size limit was thought to arise from the lipid:polymer ratio (10:1) and the microfluidic set-up used (syringe pump). Lower sizes may be achieved in the future by using a higher concentration of polymer (lowering particle/buffer interfacial tension) and replacing the oscillatory syringe pump with a pressure-driven system (generating stable laminar flow regimes at higher FRR values).

The potential instability of LLC particles at biologically relevant temperatures was assessed using DLS analysis, before and after incubation at 37 °C for 24 h. Negligible variations were seen, implying the nano-assemblies remained intact with no sign of aggregation (Fig. 2C and D for gaussian distributions and PDI values respectively). Particles were stable by DLS for up to 30 days at room temperature (see supplementary information Fig. S.4).

MHF size control is ultimately governed by the relative concentrations of nucleating species, finely tuned via the hydrodynamic diffusion gradient^30,31^. Thus, a comparison of our method with probe sonication was carried out, using lipid films of equal *end* concentrations to MHF samples. With sonication, no clear relationship between lipid concentration and particle size was observed, and PDI were largely above 0.2, with each sample tested in triplicate (Fig. S.5). While this was far from an exhaustive exploration of the full capability of high energy shearing methods, it was taken as a benchmark study to highlight the advantages of MHF over sonication.

### Cryo-transmission electron microscopy

Cryogenic transmission electron microscopy for nanoparticle analysis has been particularly useful in the study of liquid lyotropic mesophases. High resolution micrographs portray internal ordering and further image processing via fast Fourier transform (FFT) analysis can be a powerful complementary technique alongside x-ray scattering for phase assignment (FFTs matching reciprocal lattice points). Literature examples where cryo-TEM was used to analyse LLC particles served as reference points against which our data were compared^32,33^. This allowed preliminary phase assignment and estimation of lattice parameters, later validated by SAXS^32^.

Samples were generated by MHF using an FRR value of 20 (TFR 420 µL/min), concentrated for cryo-TEM analysis using centrifugal filters with 30 kDalton pores for 10 min at 10 k r.c.f. The viscous, opaque filtrates were then analysed by DLS for any signs of instability/aggregation. It was found that particles could tolerate being concentrated 100-fold before showing signs of aggregation (generally at around 400 nm). It was presumed that below this point particles merely agglomerated and could be easily re-dispersed.

**Figure 3:**
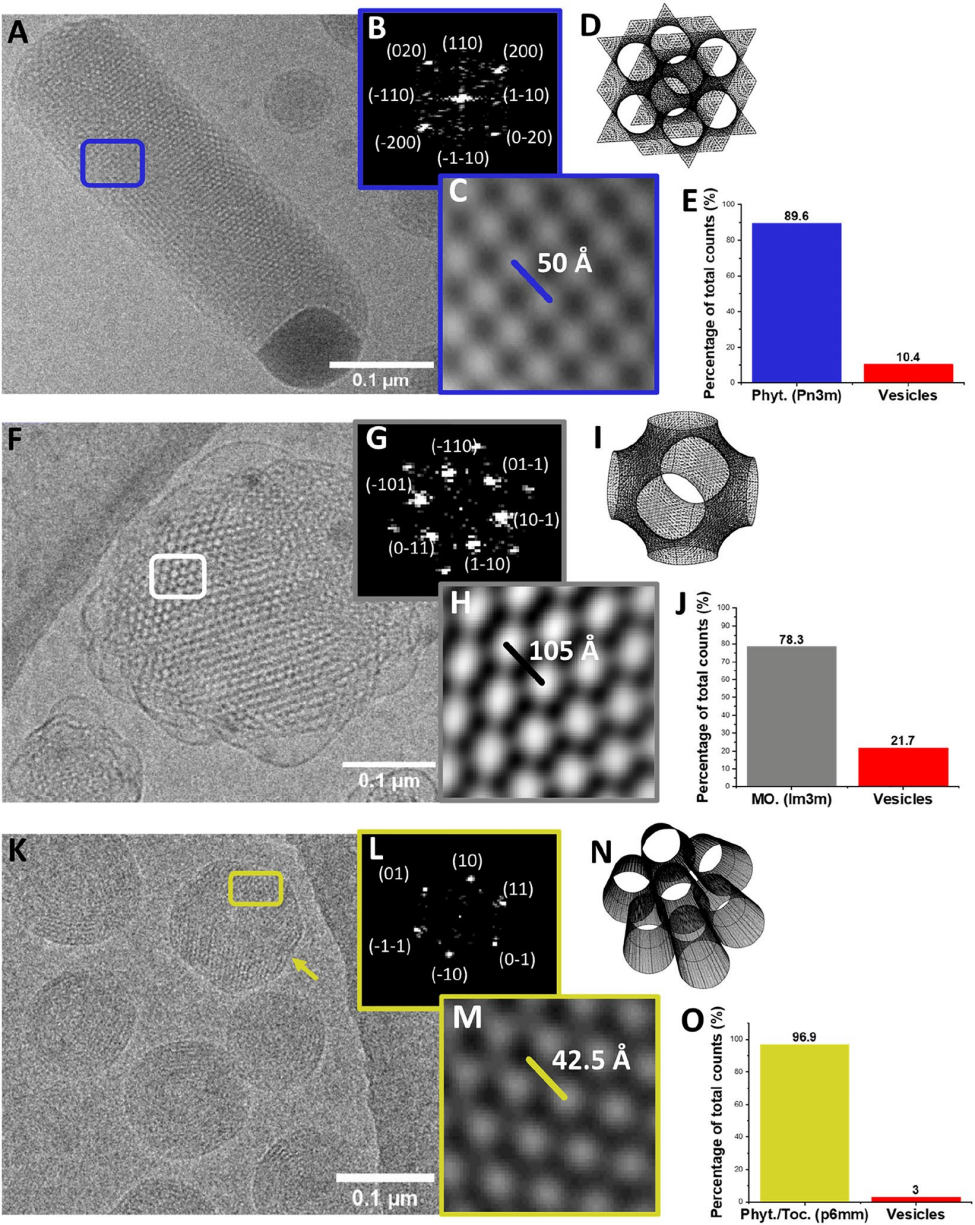
Cryo-EM and accompanying FFT analysis of LLC particles stabilised by Pluronic F-127. (**A**) Electron micrograph of a rod-shaped phytantriol cubosome. Scale bar indicates 100 nm. (**B**) Fast Fourier transform with assigned miller indices for the section marked with a blue rectangle. The square reflection pattern would indicate this section is orientated such that the [001] direction is parallel to the optical axis. (**C**) Inverse FFT of the inset FFT, with which d_110_ could be estimated via a grey scale plot taken along the blue line, and subsequently the lattice parameter a (50 Å and 70.7 Å respectively, suggesting an Pn3m phase). (**D**) Wireframe graphic showing a portion of a Schwarz double diamond minimal surface, onto which a lipid bilayer is draped for an Pn3m phase. (**E**) Percentage of cubosomes vs. lipid vesicles as estimated by cryo-EM (total particle count = 200). (**F**) Micrograph of a monoolein cubosome, displaying multiple facets and a bleb-like lamellar exterior. (**G**) FFT with assigned miller indices for the section marked in grey, the hexagonal pattern indicating a [111] direction parallel to the optical axis. A second set of reflections can be seen, in a √2: √4 ratio indicating an Im3m phase. (**H**) iFFT of the inset, with which the d_110_ and a could be estimated (105 Å and 148.5 Å respectively). (**I**) A wireframe graphic of a primitive minimal surface unit cell (Im3m). (**J**) Percentage of cubosomes vs. lipid vesicles as estimated by cryo-EM (total particle count = 150). (**K**) Micrograph of phytantriol/ tocopherol acetate (10 mol%) hexosomes. A characteristic “spine” is noted with a yellow arrow. Scale bar indicates 100 nm. (**L**) FFT of the section marked with a yellow rectangle, with a hexagonal pattern with assigned indices as before. (**M**) a was estimated at 50 Å giving a d_10_ of 42.5 Å. (**N**) Wireframe graphic of hexagonally packed tubes with 2D symmetry, representative of monolayer lipid tubules present for H_ll_ (p6mm). (**O**) Percentage of hexosomes vs. lipid vesicles as estimated by cryo-EM (total particle count = 250). All images were subject to Gaussian blur and contrast adjustments using ImageJ software. Additional images can be found in Supplementary information Figs. S.6, S.7, and S.8.

A representative example of a phytantriol cubosome is shown in Fig. 3A. The surfaces of these particles presented as tightly woven membranes close to a highly ordered particle centre. In general, particles were well-defined at their edges, and their overall shape seemed to show a partial size dependency, with smaller particles adopting a spherical shape and larger particles more faceted/crystalline. It was presumed that morphological heterogeneity reflected various stages of particle growth. Occasionally, rod-like morphologies were observed. Fast fourier transform analysis of a section of the particle shown in Fig. 3A (marked in blue) gave a characteristic cubic pattern to which miller indices were assigned (Fig. 3B). From iFFT analysis (Fig. 3C), d-spacing was estimated to be 50 Å, with a lattice parameter (*a*) ~ 70 Å. Both values were consistent with literature examples of a phytantriol/F-127 system, and indicated a Pn3m space group (several unit cells of which are shown in a wireframe graphic in Fig. 3D)^34^. It was noted that phytantriol-based cubosomes could not withstand the same electron beam intensity without loss of resolution compared to monoolein cubosomes, most likely due to their smaller interplanar spacing. For monoolein-based cubosomes stabilised by Pluronic F-127, samples were considerably more turbid compared to similarly sized LLCs of other compositions. As is shown in Fig. 3 F, particles adopted a characteristic petal-like morphology, with membrane blebs encompassing a highly ordered inner core. Several crystallographic directions were observed in the particle shown, suggesting a partially faceted structure. The section marked in white was subjected to FFT analysis and miller indices were assigned as before, this time for a hexagonal motif suggesting a [111] direction parallel to the optical axis (Fig. 3G). For monoolein cubosomes, the triblock stabilising polymer F-127 is known to influence phase behaviour (hydrated monoolein preferentially forms a Pn3m system in the absence of F-127)^34–36^. A secondary set of FFT points was observed. This proved useful in preliminary phase assignment as the first and second set of points were present in a √2: √4 ratio (suggesting Im3m). Figure 3H (iFFT) was used to estimate the interplanar distance (d-spacing) and lattice parameter (*a*): ~ 105 Å and 148.5 Å respectively. These values were in excellent agreement with those previously reported for similar lipid/polymer compositions reporting an Im3m space group (a wireframe unit cell is shown in Fig. 3I)^12,36^. Finally, phytantriol particles doped with tocopherol acetate (10 mol%) were analysed. A representative micrograph is shown in Fig. 3K. Differentiating between cubosomes and hexosomes via electron microscopy was non-trivial. Both inverse bicontinuous cubic particles seemed to preferentially orientate such that [111] directions were perpendicular to the sample plane, producing hexagonal FFT patterns. It was therefore important to establish that: (a) cubic FFT patterns could be found in the two cubic systems^32^, (b) a characteristic ‘’spinning top’’ like morphology could be observed in the presumed hexosomal samples^37^, and (c) estimated lattice parameters were in agreement with literature values. All three criteria were met, and hexosomal particles with d_10_ of approximately 42.5 Å were obtained and analysed via FFT and iFFT analysis (Fig. 3L,M). A crest can be observed near the apex of a particle marked with a yellow arrow in Fig. 3K, identical to those described by Dong et. al., who use a similar composition^37^. Various spiral particles resembling snail shells were also observed, reminiscent of toroidal DNA condensates. These were most likely the same ‘’spinning-top’’ structures viewed down another optical axis^37–39^. A wireframe graphic depicting the hexagonal packing of cylindrical tubes (characteristic of an inverse hexagonal phase) is shown in Fig. 3N. In addition to preliminary phase assignment, cryo-TEM was used to measure the percentage of lamellar vesicles (single bilayer spheres) formed alongside LLCs. This has been noted in the past, particularly for methods involving ethanol evaporation (as much as 50% vesicles can be present in samples composed of phytantriol, described in full by Akhlaghi et al.)^32^. Up to 40 cryo-EM micrographs and 200 particles were counted for each particle class generated by MHF. There was a clear bias for the formation of LLCs over vesicles based on these results (shown in Fig. 3E,J,O), confirming the appropriateness of this method over others for future studies highly dependent on particle morphology.

### Small angle X-ray scattering (SAXS)

To confirm the phase behaviour of each particulate system, X-ray scattering techniques were employed, diffraction patterns yielding key information on LLC crystallinity. Lipid pastes are typically required for SAXS analysis (very low water content). As a result, MHF samples were concentrated using centrifugal filters as previously described. Samples were analysed before and after concentrating by DLS to assess degree of particle aggregation, though this was not expected to drastically alter scattering.

Relating the Ewald sphere to Bragg’s law, the scattering vector *S*_*hkl*_ can be expressed in terms of the interplanar spacing of the crystal lattice (*d*_*hkl*_ where *hkl* refer to miller indices of a given plane), and the *q*_*hkl*_ value obtained experimentally (position of Bragg peak), with:

2 $${S}_{hkl}=\frac{{q}_{hkl}}{2\pi }= \frac{1}{{d}_{hkl}}$$

Lattice parameters were then calculated with respect to each assigned crystalline space group for cubic systems Pn3m and Im3m, and plane group p6mm for inverse hexagonal (H_ll_) systems using Eqs. (3) and (4) ^40–42^.

3 $${a}_{cubic}=\frac{2\pi \sqrt{{h}^{2}+{k}^{2}+{l}^{2}}}{{q}_{hkl}}$$

4 $${a}_{hexagonal}= \frac{4\pi \sqrt{{h}^{2}+{k}^{2}-hk}}{\sqrt{3}{q}_{hk}}$$

**Figure 4:**
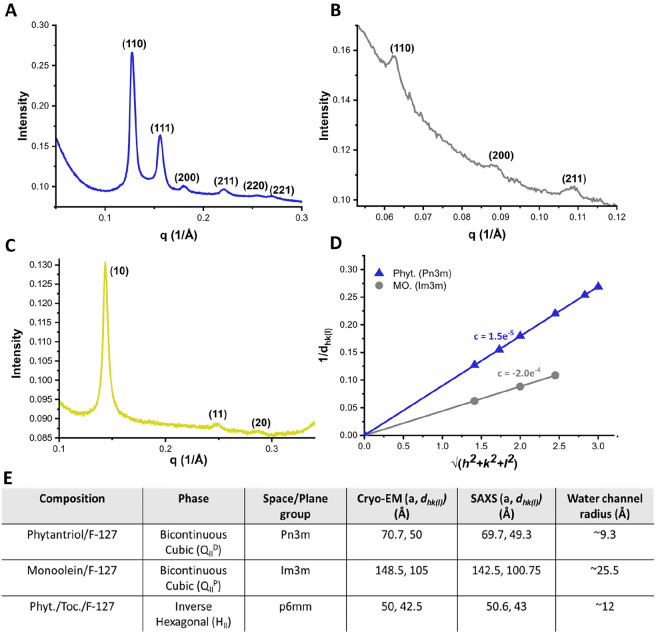
SAXS results with a table comparing lattice parameters (a) and d-space values to those calculated via FFT analysis of cryo-EM micrographs. (**A**) Bragg peaks for phytantriol samples were present in the ratios: √2: √3: √4: √6: √8: √9, indicative of a Pn3m bicontinuous cubic phase, with corresponding miller indices (110), (111), (200), (211), (220), (221). (**B**) Monoolein samples gave peaks with: √2: √4: √6 (indicative of Im3m bicontinuous cubic) with miller indices: (110), (200), (211). (**C**) Finally, peaks for phytantriol with tocopherol acetate (10 mol%) were present as: 1: √3: √4 (indicative of an inverse hexagonal phase, with plane group p6mm) with miller indices (10), (11), (20). (**D**) Graph illustrating the linear relationship between miller indices and experimentally obtained q values. Intercepts of a linear fit for each data set is shown as very near 0, indicating correct phase assignment. (**E**) Table comparing SAXS and cryo-EM derived lattice parameters and d-spacings.

Bragg peaks with miller indices are shown in Fig. 4A–C, corresponding to phytantriol (blue), monoolein (grey) and phytantriol/tocopherol acetate (yellow). Linear regression plots are given in Fig. 4D. The intercept of the regression line for phytantriol and monoolein cubosomes was very near zero, signifying correct phase assignment. A table in Fig. 4E compares *a* and *d*_*hk(l)*_ values obtained via SAXS with those estimated by cryo-EM. All values were in excellent agreement with one another.

It was then possible to estimate the radii of water channels for each phase, using the SAXS derived lattice parameters (*a*), approximate lipid chain lengths (*l*_*c*_) (with C–C bonds taken as ~ 1 Å for lipids in a liquid disordered state), and Eqs. (5) and (6), derived by Briggs et al.^43,44^:

5 $${{{r}_{w}}^{Pn3m}=a\left(0.391\right)-l}_{c}$$

6 $${{{r}_{w}}^{Im3m}=a\left(0.305\right)-l}_{c}$$

*L*_c_ for monoolein (C _18_) was approximated as 18 Å, with phyatantriol as ~ 13 Å. To estimate *r*_*w*_^*Hexagonal*^*,* the following expression [Eq. (7)] was used:

7 $${{{r}_{w}}^{Hexagonal}=\frac{a}{2}-l}_{min}$$

This was derived by considering the geometry of hexagonally close-packed lipid tubules that characterises the inverse hexagonal phase, with *l*_*min*_ = *l*_*c*_ (~ 13 Å for phytantriol)^42^. Using the values outlined in Fig. 4E, water channel radii were calculated to be 9.3 Å, 25.5 Å, and 12 Å for phytantriol cubosomes, monoolein cubosomes and phytantriol/tocopherol acetate hexosomes respectively.

### Cargo encapsulation

Most LLC particles are fabricated using high energy shearing methods like probe sonication. In addition to associated costs, health risks, and batch-to-batch variability, this method can have a profoundly adverse effect on delicate biomolecular cargo, requiring encapsulation/loading steps to be performed post-formation^23–26^. To explore the potential of this on-chip method to both produce and simultaneously load LLC nanocarriers, two assays were included. A dye was used in each, curcumin (hydrophobic) in the first, drawing from a recent paper by Chang et al.^45^ and carboxyfluoroscein (hydrophilic) in the second.

### Curcumin encapsulation

Curcumin proved useful as a spectroscopic tool for measuring the hydrophobic loading efficiency of each LLC particle, with a logD value around 4 at pH 7.4 (estimated using ChemAxon). Lipid films were prepared in the presence of curcumin, at 5 mol % with respect to lipid concentration. Samples were redissolved in an appropriate amount of anhydrous ethanol and focused using an aqueous Pluronic F-127 solution, adjusting flow rates such that final lipid:polymer ratios were 10:1 as before (FRR 10). Three separate experiments were carried out for each composition and compared to controls where no lipid was present.

**Figure 5:**
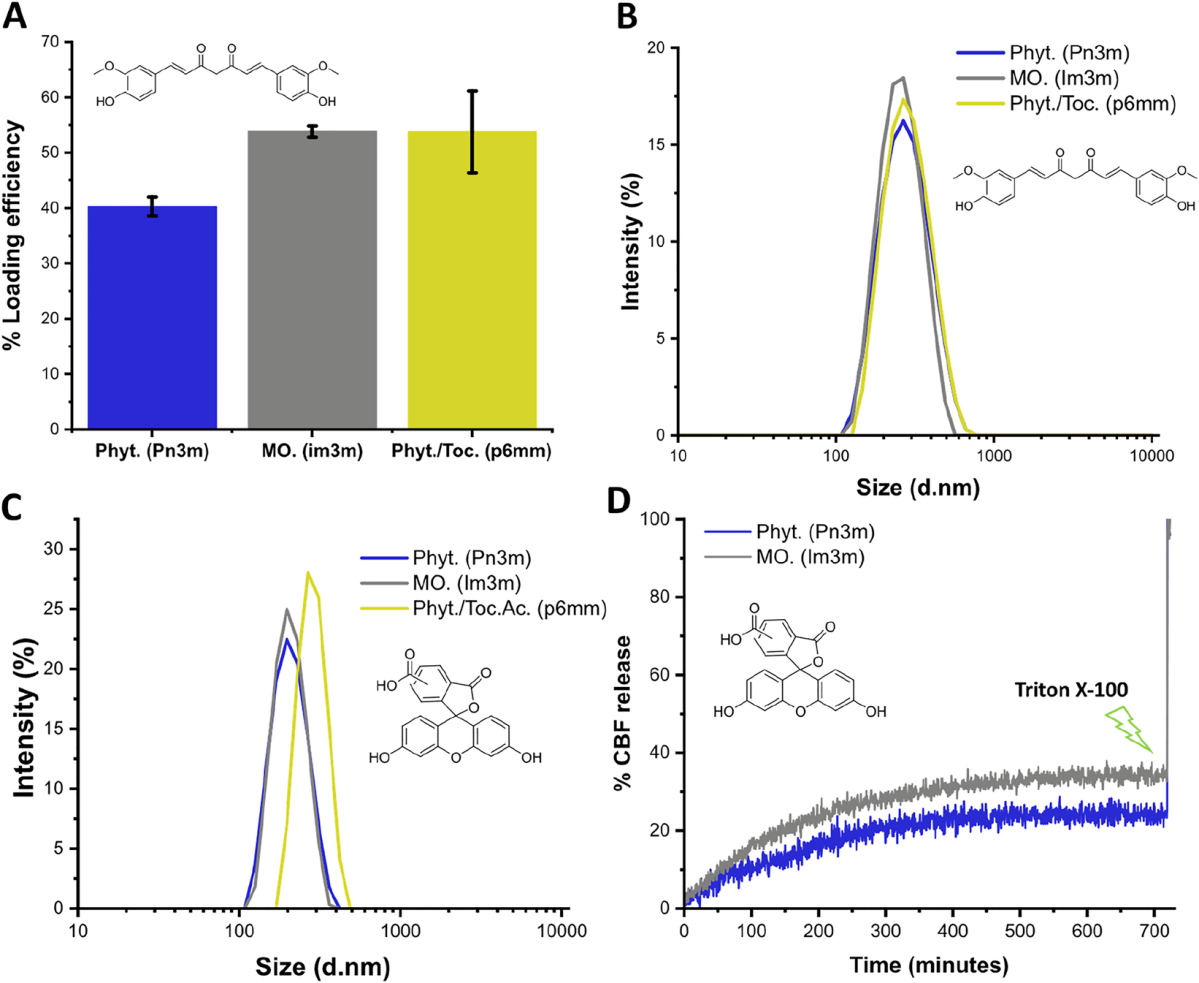
Cargo encapsulation. (**A**) % Loading efficiencies of 5 mol% curcumin (hydrophobic) in each particle type. Error bars indicate the standard deviation between three separate experiments. (**B**) Gaussian size distributions for each particle type, loaded with curcumin. (**C**) Gaussian size distributions for each particle type, loaded with CBF (hydrophilic). (**D**) A release profile of CBF encapsulated in phytantriol and monoolein cubosomes. Passive release was observed for both LLC particles over 12 h. An immediate jump in fluorescence intensity was noted after addition of Triton X-100 (causing particle lysis), demonstrating successful encapsulation of hydrophilic cargo.

Samples were left to equilibrate overnight as is described in detail by Chang et. al., to allow the water-insoluble, unencapsulated curcumin to aggregate and precipitate^45^. Samples were then centrifuged at 13 k r.c.f. for 10 min each, the supernatant (containing LLC particles) removed carefully, and the curcumin pellet redissolved in ethanol. The supernatant was analysed via DLS. Each particle population was determined to have a hydrodynamic diameter of around 250 nm. Redissolved curcumin pellets were measured for absorbance and compared to a standard curve (Fig. S.9). % Curcumin encapsulation (given as % loading efficiency) was then calculated using the difference in concentration before and after LLC formation^45^. % loading efficiencies for monoolein based cubosomes and phytantriol based hexosomes were around 50%, with phytantriol cubosomes slightly lower at around 40% (Fig. 5A). No appreciable difference in DLS-derived size distribution was seen for MHF samples before and after equilibration, with sizes and PDI values: 240 nm (0.07 PDI), 228 nm (0.14 PDI) and 217 nm (0.13 PDI) for Pn3m, Im3m, and p6mm particles respectively (Fig. 5B). It is worth noting that a % release efficiency was not calculated for curcumin as it does not self-quench, making this a difficult property to measure directly—no spectroscopic difference before and after particle micellization would be observed (via addition of a surfactant—see carboxyfluorescein and calcein loading sections for reference).

### 5(6)-carboxyfluorescein encapsulation

To investigate the loading efficiencies for hydrophilic cargo of each LLC particle, we sought to adapt a commonly used fluorescence assay involving a self-quenching dye used for liposomal systems. 5(6)-carboxyfluorescein (CBF) is quenched at high concentrations due to dimerisation and subsequent intersystem crossing. When lipid particles containing this dye are solubilised by a surfactant, the dye is released and diluted in the surrounding media, marked by a sudden increase in fluorescence intensity (demonstrated in the release profile plot in Fig. 5D).

CBF was dissolved at 50 mM in a HEPES/KCl (10/150 mM) buffer at pH 9.5. LogD values of CBF at this pH were estimated to be between − 4 and − 6 (using ChemDraw 18.2), thus ensuring the dye’s retention in the aqueous compartments of each LLC. CBF was dissolved in the ethanol stream at the same concentration. LLC particles were then prepared as before, choosing an FRR value of 10 which gave particles of 206 nm, 207 nm, and 264 nm for phytantriol, monoolein, and phytantriol/tocopherol acetate particles respectively, with PDI values 0.06, 0.05 and 0.08 for each (DLS results shown in Fig. 5C).

Particles were columned using size exclusion chromatography with Sephadex G-50, and release efficiency was estimated by comparing the fluorescence intensity before and after treatment with Triton X-100 (2.5 µL; 5% w/w) (assumed full release of encapsulated cargo). Normalised end fluorescence intensities of LLC column fractions were then compared to a standard curve for CBF in the same buffer/pH environment (See Fig. S.10A) to estimate % loading efficiencies (Fig. S.10C). The same fractions were also used to determine passive release of CBF dye at room temperature over 12 h (Fig. 5D).

Interestingly, monoolein cubosomes gave considerably higher % loading efficiency of this hydrophilic dye than phytantriol cubosomes and phyt./toc. acetate hexosomes. The Pn3m phytantriol particles showed minimal loading efficiency. Hexosomal particles, while present at the same lipid concentration and with a similar size distribution, encapsulated dye concentrations below the dynamic range of the standard curve. This result could be attributed to differences in lattice parameters/phase behaviour, with water channel volumes expected to follow the trend: Im3m cubosomes >> Pn3m cubosomes > p6mm hexosomes.

### Influence of encapsulant on internal particle morphology

Depending on encapsulant used, the internal ordering/phase of particle may be altered. For small molecules this is largely dependent on specific interactions and positioning within a lipid assembly. As such, we imaged dye-laden nanoparticles of each phase type via cryo-TEM and compared estimated *d*_*hk(l)*_ values with those of ‘empty’ particles. The results are shown in Fig. S.11 (supplementary information). CBF was expected to preferentially partition into the water channels of each LLC particle, and curcumin into hydrophobic membrane leaflets. Values for *d*_*hk(l)*_ for all particles did not change however (within at most 2 Å of measured d-spacing for empty particles). These results indicated that assigned phases were retained after dye loading, though SAXS data is needed before conclusions are made with respect to *d*_*hk(l)*_*/a*.

### Size dependant payload delivery

**Figure 6:**
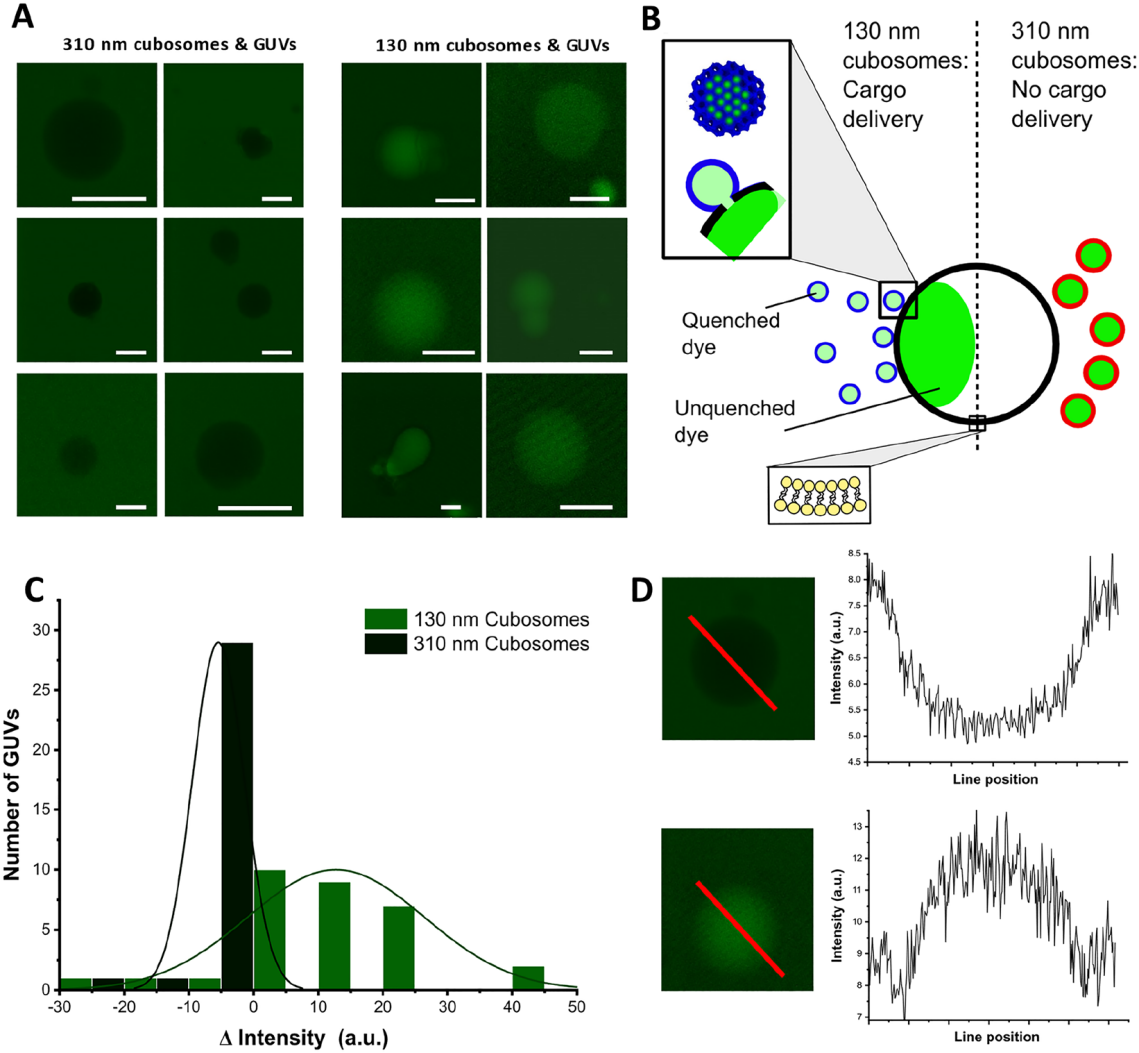
Size dependant payload delivery. (**A**) Confocal images of GUVs exposed to phytantriol-based cubosomes containing calcein (40 mM) of 310 nm (left column) and 130 nm (right column). All scale bars shown correspond to 10 micron. GUVs were on average between 8 and 20 micron in diameter. (**B**) Graphical illustration of the experiment. Structures are not to scale. (**C**) Distribution of intensity differences for calcein, measured as inner minus outer intensity. 64 GUVs measured in total, across three separate experiments. (**D**) Intensity profiles across two GUVs (both approximately 15 micron in diameter). Values used for the histogram in C were derived from profiles like these.

To showcase the importance of controlling particle size (a feature of our production method), we performed studies on the ability of phytantriol-based cubosomal particles of different sizes to fuse with model cell membranes [giant unilamellar vesicles (GUVs)]. We loaded our particles with 40 mM calcein (self-quenched) which, following successful fusion with giant vesicles and mixing of aqueous material via a fusion pore, would lead to a higher fluorescent signal in the GUV lumen. The dye was loaded into the particles via microfluidics as before, purified via SEC, and concentrated to an approximate particle concentration of 5 × 10^10^ particles/ml (determined using Multi-angle DLS). Two size populations were generated, at 130 nm and 310 nm, and diluted to adjust for any discrepancy in concentration between the two. Separately, giant vesicles composed of DOPC with PE-Rhodamine (0.5 mol%) were generated via electroformation (See Fig. S.12 for red-channel images). These were mixed at a 1:1 ratio with each LLC size class separately via gentle pipette aspiration, and transferred to a coverslip for imaging. Confocal images were taken of individual giant vesicles after at least 15 min (to allow vesicles to settle). The green channel (detecting fluorescence between 500 and 530 nm) was used to determine whether calcein had been delivered to the aqueous centre of the GUV, without loss of membrane integrity, marked by a significant increase in fluorescence intensity (representative results are shown in Fig. 6A). The intensity difference (ΔI) between the inside and outside of each GUV was calculated: a positive value defining a positive delivery event, a negative value indicating no calcein delivery. Intensity differences were plotted for each cubosome size class in the histogram shown in Fig. 6C. The distribution of ΔI was assumed to be a function in variation in size of giant vesicles (generally between 8 and 20 μm—a consequence of electroformation methods). In future work, droplet templating approaches such as the octanol assisted liposome assembly (OLA) technique may prove useful in eliminating polydispersity with regard to GUV size^46^. Examples of how intensity profiles were used in calculating ΔI are shown in Fig. 6D. A total of around 10 GUVs for each LLC size class were analysed, and repeated in triplicate (histogram shows 64 GUVs in total). A marked difference was noted between size classes. Particles at 130 nm were seen to deliver calcein, whereas 310 nm particles did not. No obvious change in GUV membrane morphology was observed in either case (see Fig. S.12).

No significant incubation time was needed for calcein delivery to occur (calcein containing GUVs observed immediately after population mixing), potentially indicating a rapid fusion process. Future work involving microfluidic GUV trapping experiments may be useful in extracting precise kinetic information. In a seperate experiment, cubosomes (130 nm) were doped with 0.5 mol% PE-Rhodamine B. Fluoresence intensity corresponding to these cubosomes was observed to accumulate around the GUV membrane (See Fig. S.13), indicating some form of positive interaction.

While the exact mechanism for such spontaneous fusion (depicted in Fig. 6B) is unkown, the relationship between particle size, curvature, and propensity to fuse with a bilayer may be explained via computational modelling, similar to what has been presented by W. T. Góźdź. They discuss that, while the phase assigned to particles of both size populations may be identical, smaller particles may assume several possible topological varients^47^. Bilayer deformation at the facets of each particle class is necessary for bending energy minimisation, though has been proposed as having a greater influence on smaller particles over larger ones. Therefore, a greater degree of curvature frustration may be present in particles at 130 nm, leading to a higher frequency of spontaneous fusogenic events. Additional support for this can be found in an excellent study conducted by Golani and Schwarz, who employ elastic theory to predict fusion behaviour between curved and planar membranes^48^.

## Conclusions

Following the principles of microfluidic hydrodynamic focusing, we have developed a platform for the synthesis of liquid crystalline particles. Cubosomes (both Pn3m and Im3m) and hexosomes (p6mm) were produced on-chip with exemplary size control, ranging between around 130 nm and 300 nm. This has profound implications for the field of soft-matter nanotechnology, size being a critical parameter in determining particle end-function. The method benefits from unique advantages associated with microfluidic technologies, including low energy consumption, minimal user input, and integration of downstream processing modules. Though serpentine channels and other microfluidic chip designs could increase the efficiency of particle generation, this may come at a cost with regard to size control. Introducing secondary flow regimes (Dean flow etc.) can increase mixing efficiency between the ethanol and aqueous phases but adjusting the concentration of nucleation points via small changes in flow rates may be more difficult, compared to a linear channel where the formation of nucleation aggregates is wholly dominated by lateral diffusion at a well-defined ethanol/water interface. Future work involving alternative chip architectures will take inspiration from previously established techniques for microfluidic nanoparticle generation^3,5^. The scalability of MHF has previously been demonstrated via parallel assembly lines and aspect ratio changes in channel architecture, which will no doubt apply to this method should industry-scale synthesis be required^27,49^. Translating this work to platforms made of cheaper materials (glass capillaries and alternative plastics) could also benefit synthesis at an industrial scale, and adoption of 3D printed devices will promote wider adoption and facilitate rapid prototyping of different device designs^50,51^.

Samples were generated at rates of over 400 µL/min at 1.4 mM. Crucially, particles were stable at 37 °C and were comparable in crystallinity to those of a similar composition generated via probe sonication, without the need for elevated temperatures and high-shear conditions known to destabilise more sensitive biomolecular cargo.

The effect of ethanol (< 10% v/v) was seen to be negligible though further work may be needed to optimise the chip for removal of any residual solvent. This could likely take the form of on-chip dialysis, as has already been demonstrated by Hood et. al. for liposomal formulations^5,52^.

Finally we have highlighted the importance of size control in determining downstream applications for phytantriol-based cubosomes. Successful delivery of a self-quenching dye to GUVs was observed with smaller nanoparticles, presumably via membrane fusion, whereas larger particles were incapable of dye delivery.

We have successfully demonstrated the on-chip generation of LLC particles (cubosomes and hexosomes), though the technique could likely be expanded to include other soft-matter architectures (micellosomes, multicompartment nano-vesicles etc.) via compositional adjustments. This opens the possibility of rapid on-chip prototyping of higher-order assemblies in an array-like fashion. As a result, this platform has significant potential in aiding modern medicine, particularly vaccinology, where research is turning ever toward these specialised nano-carriers.

## Materials and methods

### Microfluidic chip fabrication

Chips capable of microfluidic hydrodynamic focusing were designed using AutoDesk360 (See Supplementary Information Fig. S.3A). Initial designs were loosely based on commercial Dolomite chips used in MHF liposome synthesis. They featured a flow focusing junction made up of three channels: two with a shared inlet and one central channel with a separate inlet. Channels adjacent to the central channel were kept at 45°. Pre-junction dimensions were equalised by adding a serpentine section in the central channel, and the total length roughly covered that of a microscope slide (70 mm). With a view toward introducing secondary flow regimes, serpentine channels were added near the outlet in some later designs but are not shown here. CAD designs were sent to Micro Lithography Services Ltd. (Chelmsford CM3 5ZF, UK) for laser cut photolithography masks. In a cleanroom, under UV filtered light, SU-8 2075 (Kayaku Advanced Materials Inc., Westborough, MA 01581 USA) was deposited onto a silicon wafer by spin coating at a specified rpm using a spin coater (WS-650-8 model, Laurell Technologies Corporation, North Wales, PA 19454-4150 USA). The wafer was subjected to a pre-exposure “soft bake” on a heating mantle. The laser-cut mask was placed on the wafer and exposed to UV light (UV-OAI 150), followed by a pre-development heating cycle. The master was carefully submerged in a propylene glycol methyl ether acetate (PGMEA) (Sigma Aldrich) bath to remove any excess photoresist polymer. The wafer was then treated with acetone (Sigma Aldrich). The PGMEA wash was repeated until it was evident all excess SU-8 had been removed (waste acetone ran clear rather than white). The wafer was dried under a steady stream of nitrogen, then heated at 65 °C for 10 min. Details of photoresist deposition and curing conditions required for a feature height of ~ 150 µm are shown in Table 1.

**Table 1:** Conditions used in the fabrication of 150 µm high SU-8 2075 photoresist features on a silicon wafer.

Process	Conditions used
SU-8 2075 deposition	− 500 rpm (10 s) decelerate to 100 rpm (1 min) − 1200 rpm (30 s) decelerate to 300 rpm (1 min) − 0 rpm accelerate to 400 rpm (10 s)
Pre-exposure bake	65 °C (6 min); 95 °C (12 min)
UV exposure	9.7 mV; 300 mJ/cm^2^ dose; 30s
Post-exposure bake	65 °C (5 min); 95 °C (12 min)

These conditions were derived from the manufacturing specifications for the photoresist polymer.

Features were inspected using a microscope (MZ12 module, Leica Camera AG; 2.5 × objective) for any defects. If judged to be of a satisfactory quality, the master was plasma treated for 1 min in a plasma oven (Harick Plasma oven; model: PDC-002), after which it was placed under vacuum in a desiccator for 1 h, along with a 1 ml glass vial containing trichloro(1H,1H,2H,2H-perfluorooctyl)silane (0.1 ml; Sigma Aldrich). The wafer was placed into a petri dish to await further use in PDMS chip fabrication. Vinyl-terminated polydimethylsulfoxane (PDMS) (50 g; Sylgard 184, Sigma Aldrich) was poured into a 100 ml weigh boat. The cross-linking agent (5 g) was incorporated by mixing with a plastic spatula. Some of the mixture (40 g) was poured onto the featured wafer, and the remainder into a separate petri dish. Both dishes were degassed in a desiccator under vacuum for 1 h, then placed onto a hotplate at 65 °C to cure overnight. The featured PDMS block was cut out using a scalpel, and the inlets and outlet punched using a 2 mm biopsy punch. The featured PDMS block (feature side up) and the PDMS blank were both plasma-treated for 1 min then pressed firmly together, taking care to remove any air bubbles. RainEx (hydrophobic glass treatment solution made up of a silane mixture; 0.2 ml) was injected into the channels at this stage, then immediately blown out using a nitrogen stream. Excess surface treatment was evaporated, and the bonding step completed by heating the sandwiched blocks to 65 °C for 15 min. Chips were cut to the required shape, then plasma bonded onto a glass slide. Magic tape was used placed over the surface of the chips to prevent any channel contamination before use.

### LLC generation via MHF

Lipid films were prepared by first melting the required amount in a glass vial at 60 °C. The resultant lipid film was dissolved in dry ethanol (Sigma Aldrich) by sonication at 37 kHz for 30 min (XUBA-3 model, Grant Instruments, Cambridge SG8 6GB, UK) to 14 mM. This was filtered using a syringe filter (0.45 µm pore size; polyether sulfone) into a new vial that had been blown thoroughly with a nitrogen stream for ~ 3 s. For tocopherol acetate compositions, slightly elevated temperatures were needed for adequate dissolution. Separately, the aqueous phase was prepared by dissolving Pluronic F-127 in 1 × PBS (pH 7.4) at a concentration such that lipid:polymer ratios could be easily maintained at 10:1 using a seperate diluting flow of 1XPBS adjoined via an off-chip t-junction (see Fig. S.3C for details on flow rates and concentrations used). Solutions like these were prepared on the day of MHF experiments. All solutions were taken up by plastic Normject syringes and affixed to a syringe pump (Fusion Touch 100, KR Analytical). The MHF chip was firstly charged with dry ethanol, then affixed to a clear acrylic sheet with BlueTac and placed into a microscope holder (Nikon TE 2000; 4 × objective). The syringes were connected via microfluidic tubing (PTFE, 0.8 ID × 1.8 mm OD) to their respective inlets. A piece of tubing (~ 10 cm) was fitted to the outlet of the MHF chip and fed into a 7 ml glass collection vial. The syringe pump corresponding to the lipid in ethanol solution was set to 100 µL/min and left until there was evidence of flow, as viewed through LabView software. The aqueous stream was introduced by setting the flow rate of the other syringe pump to 100 µL/min. Both streams were allowed to equilibrate (~ 1–2 min) before steadily focusing the central channel by adjusting each flow rate in 20 µL/min increments. When altering flow rate ratios (FRR), the buffer stream containing the triblock polymer was diluted appropriately using a seperate diluting buffer. Total flow rates were never increased above 480 µL/min.

### Dynamic light scattering

The approximate hydrodynamic size distributions and dispersity indices of nano assemblies formed by MHF were determined through (multi-angle) dynamic light scattering measurements. Collected samples were placed directly into a plastic cuvette and diluted 10:1 in 1 × PBS at pH 7.4, and a size distribution obtained using Malvern Zetasizer Ultra software, using refractive index values for neat monoolein and phytantriol (1.47). Software-generated correlation graphs and laser auto-adjustment values were used to measure the reliability of gaussian distributions obtained (correlogram intercepts should > 0.9).

### Cryo-TEM

Cryo-transmission electron microscopy was used to determine the size and internal ordering of LLC particles as described in results and discussion sections. Samples (3.5 µL; ~ 10 mg/mL) were transferred onto a carbon grid (Quantifoil™ R 2/2 on 300 copper mesh; Jena Bioscience), in a Vitrobot (ThermoFisher) at 95% humidity. Sample grids were blotted (blot time: 2 s; blot force: − 2; wait time: 30 s) then immediately plunged into liquid a ethane bath within a liquid nitrogen reservoir. The grid was carefully transferred to a grid holder contained within the reservoir and kept at below—180 °C. Grids were transferred under liquid nitrogen to an electron microscope sample holder (CryoEM Transfer holder, Gatan; Model 626). Defocus values (generally between − 0.5 and – 5 µm) were determined elsewhere on the grid to avoid sample damage from the electron beam. Instrument and camera used: FEI Tecnai12 bio twin 120 kV with TVIPS XF416 4K CMOS detector. Image handling and analysis was carried out using ImageJ software. Samples were concentrated 100-fold using Amicon centrifugal filter units at rcf. 30 k for 10 min (Amicon® Ultra-4 Centrifugal Filter Unit).

### Small angle X-ray scattering (SAXS)

Particulate samples were concentrated to 10 mg/mL and loaded into polycarbonate capillaries, then sealed with parafilm. SAXS data were obtained for each composition using beamline I22 at Diamond Light Source (Oxford, UK). Diffraction patterns were collected using an X-ray energy of 12 keV (wavelength 1 Å) and a sample—detector distance of 3695.43 mm. The raw data were treated using the protocol developed by beamline scientists at Diamond^53^. Lattice parameters were calculated from generated Bragg peaks and phase assignments were made using the method described in the results and discussion section.

### Hydrophobic cargo encapsulation: curcumin

Protocol for determination of % loading of curcumin in LLCs largely followed the protocol developed by Chang et al.^42^, with some minor modifications for use in microfluidics. Briefly, lipid films were made as before, with 5 mol% curcumin, and LLCs generated as before. Samples were compared to a control sample i.e. the same curcumin concentration without lipids. Samples were left to equilibrate overnight before centrifuging at 13 k r.c.f. for 10 min to generate a pellet of curcumin. Pellets were washed and centrifuged three times using 1 × PBS at pH 7.4, before re-dissolving in ethanol 1 mL. Absorbance values for each pellet at 426 nm were compared to a standard curve (shown in Fig. 5) to determine end curcumin concentrations. % Loading efficiencies were then calculated using the below expression:

$$\% Loading \, efficiency=1-(\frac{{C}_{0}-{C}_{f}}{{C}_{0}}x 100 \%)$$

### Hydrophilic cargo encapsulation: 5(6)-carboxyfluorescein

LLC particles loaded with 5(6) carboxyfluorescein were prepared using the standard MHF set-up as previously described, with both the ethanol (lipid at 14 mM) and aqueous (Pluronic F-127 in HEPES/KCl (10/150 mM) at pH 9.5) streams containing 50 mM CBF. Samples were loaded directly onto a size exclusion column (250 µl loading volume; Sephadex G-50 hydrated by HEPES/KCl buffer) and eluted as 300 µl fractions. DLS was used to determine which fractions contained LLCs (found consistently to be column fraction 6 i.e. after 1800 µL eluent buffer had been added to the loaded column), along with a release assay, where Triton X-100 (5 wt%; 2.5 µL) was added to 200 µL of each column fraction. The characteristic fluorescence intensity increase as free CBF is released from the LLCs was compared. Fluorescence was analysed using a 96-well plate in a CARY Eclipse UV–Vis/Flu. Spectrometer (Agilent), with λ_ex_ = 492 nm; λ_em_ = 517 nm and PMT value = 600 V. Normalised end fluorescence values were compared to a standard curve for carboxyfluorescein to determine % CBF encapsulation.

### Giant unilamellar vesicle (GUV) preparation

A mixture of DOPC and PE-Rhodamine B lipids (1 mg/mL; 99.5:0.5 mol% respectively) in chloroform was pipetted onto the conductive side of an ITO coated slide (~ 100 μL). This was spread using a cover slip to create a lipid film, which was further dried in a desiccator overnight. A PDMS spacer was fixed around the lipid film, which was hydrated with a PBS/Sucrose buffer (0.5 M; 0.5 mL). A second ITO slide was placed over the spacer such that its conductive side faced toward the lipid film. Two clips were attached to seal the slides, and electrodes connected to a generator placed on each. The sandwich was inverted (lipid film facing down) and heated to 65 °C. The voltage was set to 1.5 V and the amplitude set to 10 Hz for the first 2 h, then at 2 Hz for the final hour. The resultant GUVs were decanted into an Eppendorf to await further use.

### Confocal imaging

Sample mixtures of GUVs and cubosomes were placed onto a passivated coverslip (passivated with Bovine Serum Albumin) and imaged using a Leica TCS SP5 II inverted confocal microscope. Images were processed further using ImageJ software.

### Supplementary Information

Supplementary Figures.

## Data Availability

The datasets used and/or analysed during the current study are available from either corresponding authors on reasonable request.
