# Supplementary material for: A microfluidic platform for the controlled synthesis of architecturally complex liquid crystalline nanoparticles
Source: Sci Rep. 2023 Aug 4;13:12684. doi: 10.1038/s41598-023-39205-3 (PMC10403506; doi:10.1038/s41598-023-39205-3)
Supplement: Supplementary file 1 — Supplementary Figures. [file 41598_2023_39205_MOESM1_ESM.pdf]

A microfluidic platform for the controlled synthesis of architecturally complex liquid crystalline nanoparticles

Colin P. Pilkington*[a,b], Claudia Contini[b], Joseph D. Barritt[c], Paul A. Simpson[d], John M. Seddon[a] and Yuval Elani*[b]

Supplementary Information

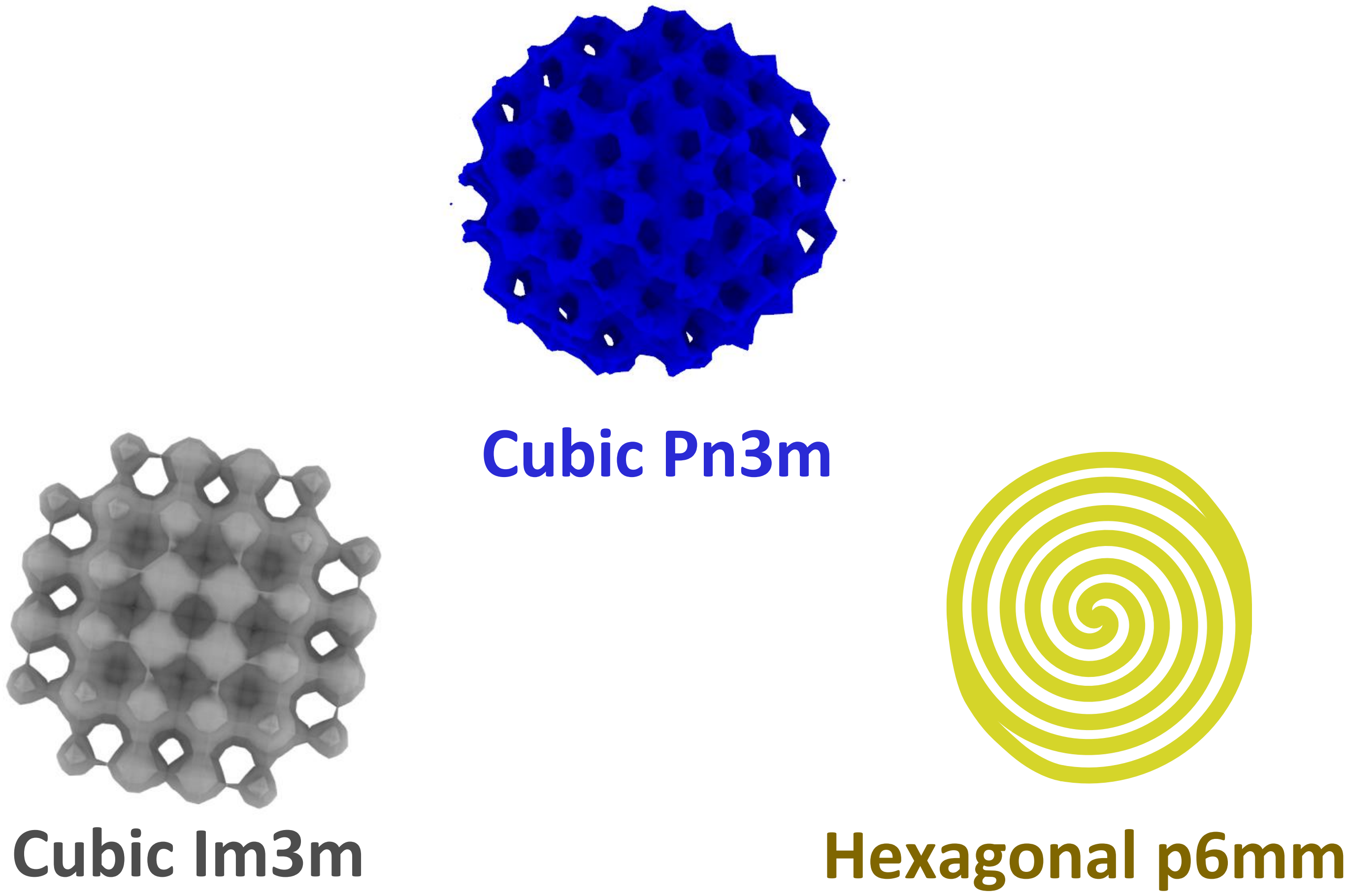

Figure S.1. *Graphics depicting each class of lyotropic liquid crystalline (LLC) nanoparticle generated. Im3m and Pn3m have symmetry in three dimensions, whereas inverse hexagonal particles have 2-D symmetry, with plane group p6mm.*

a. Department of Chemistry, Molecular Science Research Hub, Imperial College London, 82 Wood Lane, London, W12 0BZ, UK
b. Department of Chemical Engineering, Exhibition Road, Imperial College London, London, SW7 2AZ, UK
c. Department of Life Sciences, Imperial College London, Exhibition Road, London SW7 2AZ, UK.
d. Centre for Structural Biology, Department of Life Sciences, Imperial College London, Exhibition Road, London SW7 2AZ, UK.

**To whom correspondence may be addressed. E-mail: cpp19@ic.ac.uk and/or y.elani@imperial.ac.uk*

Dynamic Light Scattering – Gaussian distributions and correlograms

In general, samples were analysed via DLS at a 10:1 dilution with an appropriate buffer. This ensured adequate concentration (0.025 mg/ml end lipid conc.) for back-scattered light detection. Refractive index was set at 1.47 for both monoolein and phytantriol (values for neat lipid RI). Only gaussian distributions with correlograms of y-intercepts between 0.85 and 1 were quoted (a sanity-check for the statistical reliability of each result). Each measurement was taken in triplicate to account for any aggregation/sample heterogeneity. Mult-angle dynamic light scattering was used to deconvolute two or more size populations. This was generally only necessary where DLS results were inconclusive. The technique combines back, forward and side scattering information and Mie theory to relate scattering angles to size populations. Representative gaussian distributions and associated correlograms for measuring size variation with FRR are shown below.

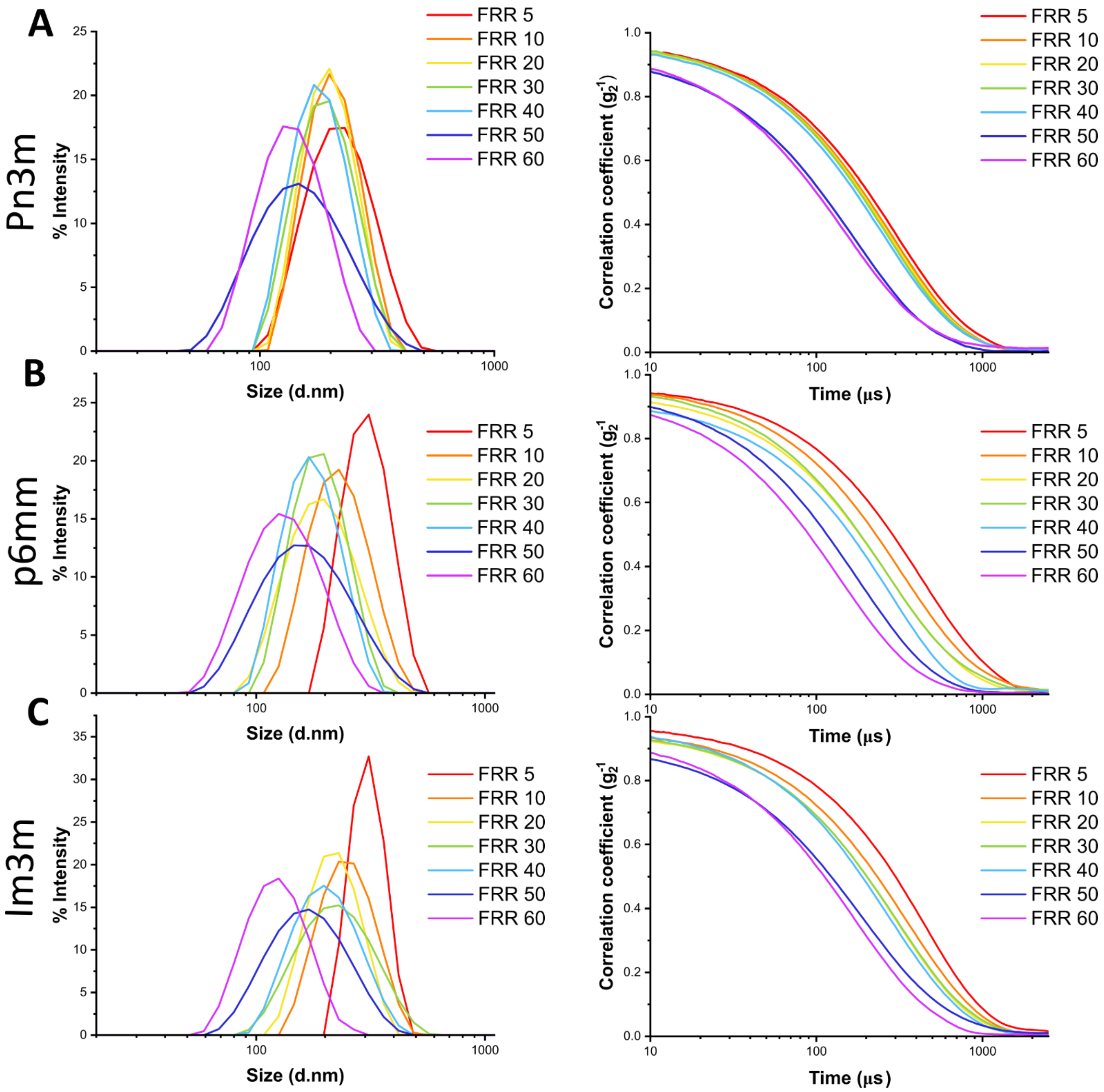

Figure S.2. ***A, B,*** *and* ***C*** *are DLS results for phytantriol cubosomes, phytantriol/tocopherol acetate hexosomes and monoolein cubosomes respectively. This data is summarised in the main text (figure 2). There is an inverse relationship between FRR value and hydrodynamic radii, which can be observed both by the gaussian plots (left) and associated correlograms (right).*

Chip design, particle stability, and back pressure measurements

The chip was designed to mimic a commercially available device consisting of a simple cross-flow junction suitable for liposome production. Flanking channels were set at 45 ° to minimise "cusping" of the central lipid stream, known to affect size distributions of generated nanoparticles at higher flow rates.[5,31,52] The PDMS mounted chip was treated with RainEx, a mixture of hydrophobic silanes, to prevent wetting of the central stream. However negligible change with respect to PDI and size distributions was detected between treated and untreated chips. Back pressure, or the pressure difference between the inlet and outlet, was measured using the Hagen-Poiseuille equation for an incompressible Newtonian fluid, given by:

$$\Delta p = \frac{8\pi\eta LQ}{A^2}$$

Where Δp is the pressure difference, η the viscosity of the fluid (taken here as 1 cP for water), L = channel length, Q = flow rate, A = cross sectional area of the channel. Back pressures around 150 mbar were present in this system.

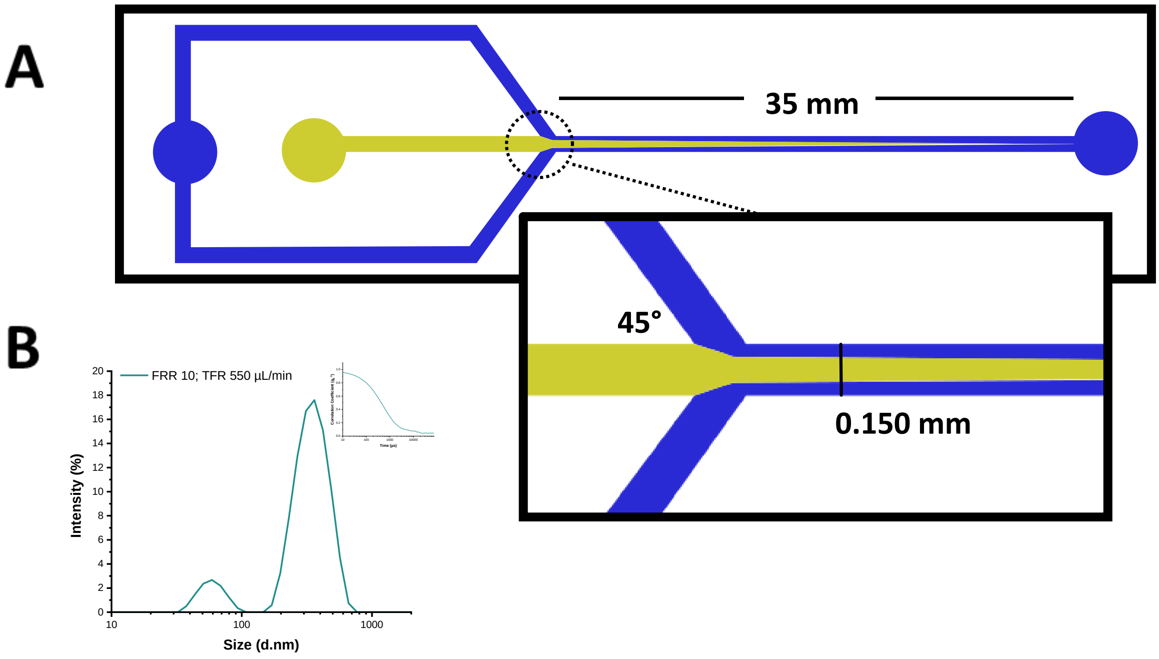

C

Flow rate ratio	Lipid flow rate (µL/min)	Diluting buffer flow rate (µL/min)	F-127 buffer (0.28 mM) flow rate (µL/min)	Total flow rate (µL/min)	End lipid concentration (starting with 14 mM of lipid) (mM)	Lipid:F-127 ratio
**5**	80.0	0	400.0	480.0	2.80	10:1
**10**	40.0	200.0	200.0	440.0	1.40	10:1
**20**	20.0	300.0	100.0	420.0	0.70	10:1
**30**	13.3	346.0	64.0	413.3	0.46	10:1
**40**	10.0	350.0	50.0	410.0	0.35	10:1
**50**	8.0	360.0	40.0	408.0	0.28	10:1
**60**	6.7	367.0	33.0	406.7	0.23	10:1

Figure S.3. ***A.*** *CAD design of MHF chip, with a zoomed view of the cross-flow junction inset.* ***B.*** *Typical DLS results at higher total flow rates (ie. >500 µL/min). Emergence of a secondary peak could indicate formation of vesicles and/or micellar structures in addition to the expected distribution around 300 nm. Oscillatory flow in the central stream was noted and presumed to be a result of increased back pressure combined with low flow rates of the central stream.* ***C.*** *Summary table of flow rates and lipid concentrations used in each experiment.*

Nanoparticle storage stability

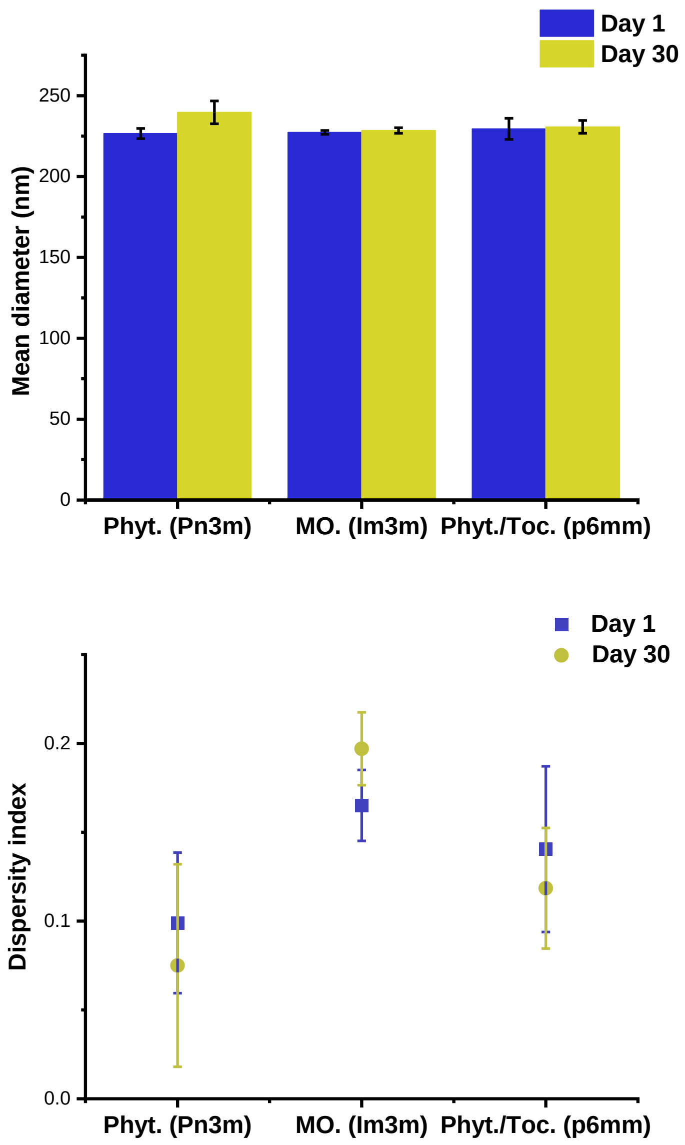

Figure S.4. *Particle stability (measured via DLS) before and after storage at room temperature for thirty days. Error bars indicate standard deviations between 3 separate experiments (3 separate films and microfluidic chips). No significant change in either hydrodynamic radii or dispersity indices was noted after 1 month of storage under ambient conditions.*

Probe sonication comparison

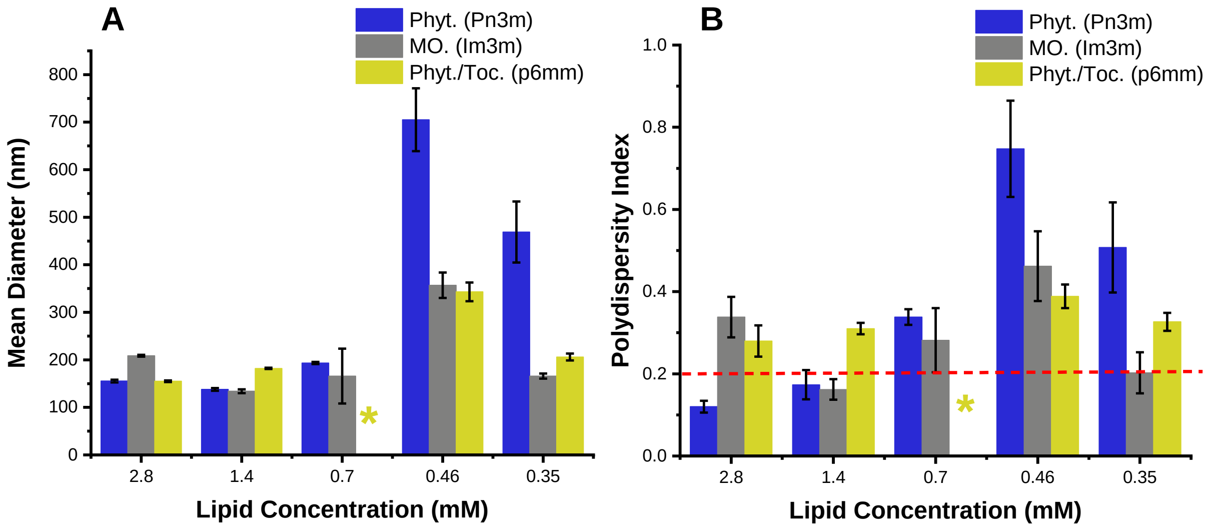

Figure S.5. *Results of DLS analysis on probe sonicated samples. Films of equal end-lipid concentration to MHF samples were hydrated in F-127 solution in 1X PBS at the appropriate concentration, such that a 10:1 ratio of lipid to polymer was maintained. Samples were then subjected to 5 minutes of ultrasonication at 40% amplitude, with 5s on 5s off pulsing. Samples were then diluted 10:1 in 1X PBS (identically to MHF samples) and analysed via DLS. 3 samples at each concentration for each composition were prepared (n = 3) and the mean particle size taken (**A**), along with the mean PDI (**B**). Error bars in both graphs indicate standard deviation for n = 3. The red dashed line in graph **B** indicates the threshold for PDI for nanoparticles suitable in industry (0.2). The purple asterix for hexosomes at 0.7 mM indicates that number fluctuations were present during DLS analysis, most likely due to insufficient particle concentration.*

Monoolein Cubosomes (Im3m) – Cryo -EM

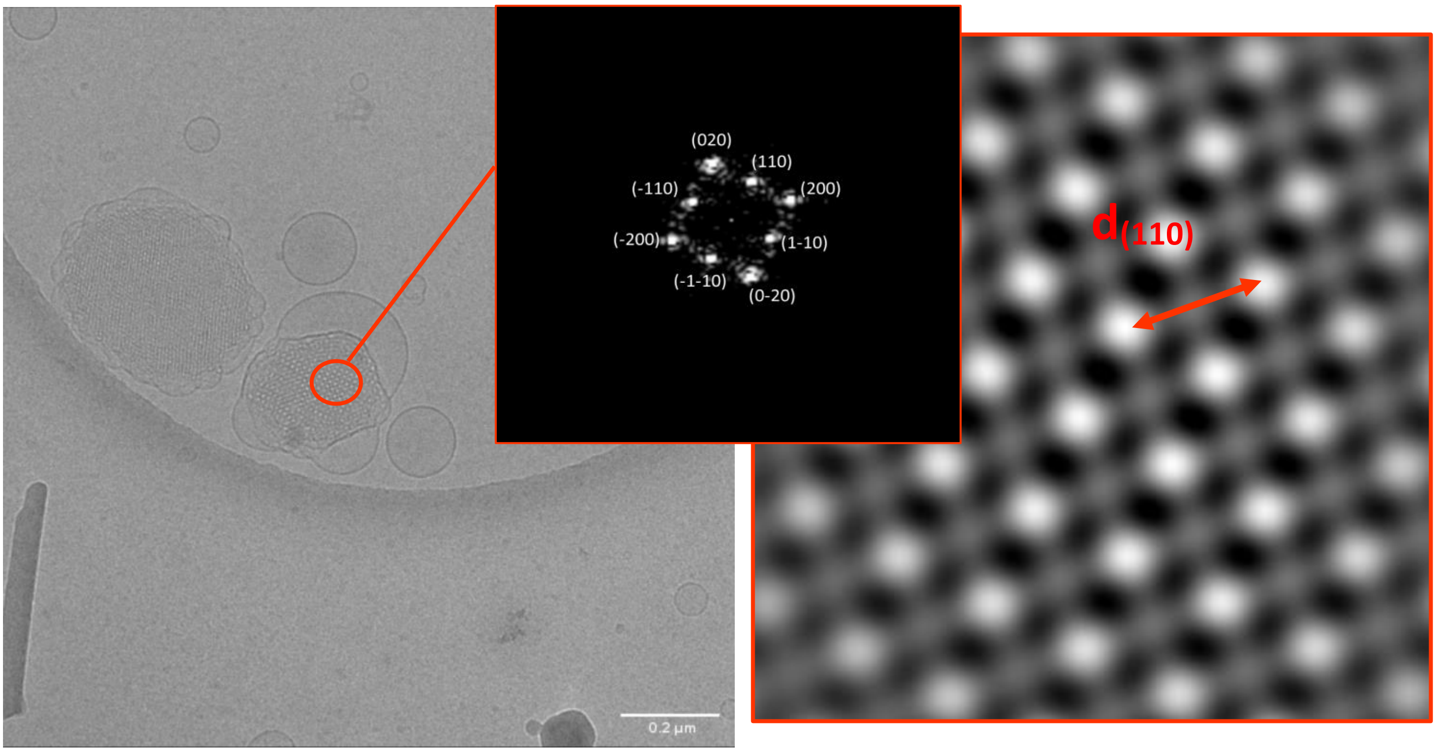

Figure S.6.: ***A*** *Supplementary cryo-EM image with insets showing FFT and iFFT of cubosomes. Square pattern shown in A FFT indicative of <001> direction down cubic lattice. Micrographs also show co-existence of vesicular particles. Scale bar indicates 200 nm. Significant blebbing of lamellar leaflet can be seen.*

Phytantriol Cubosomes (Pn3m) – Cryo -EM

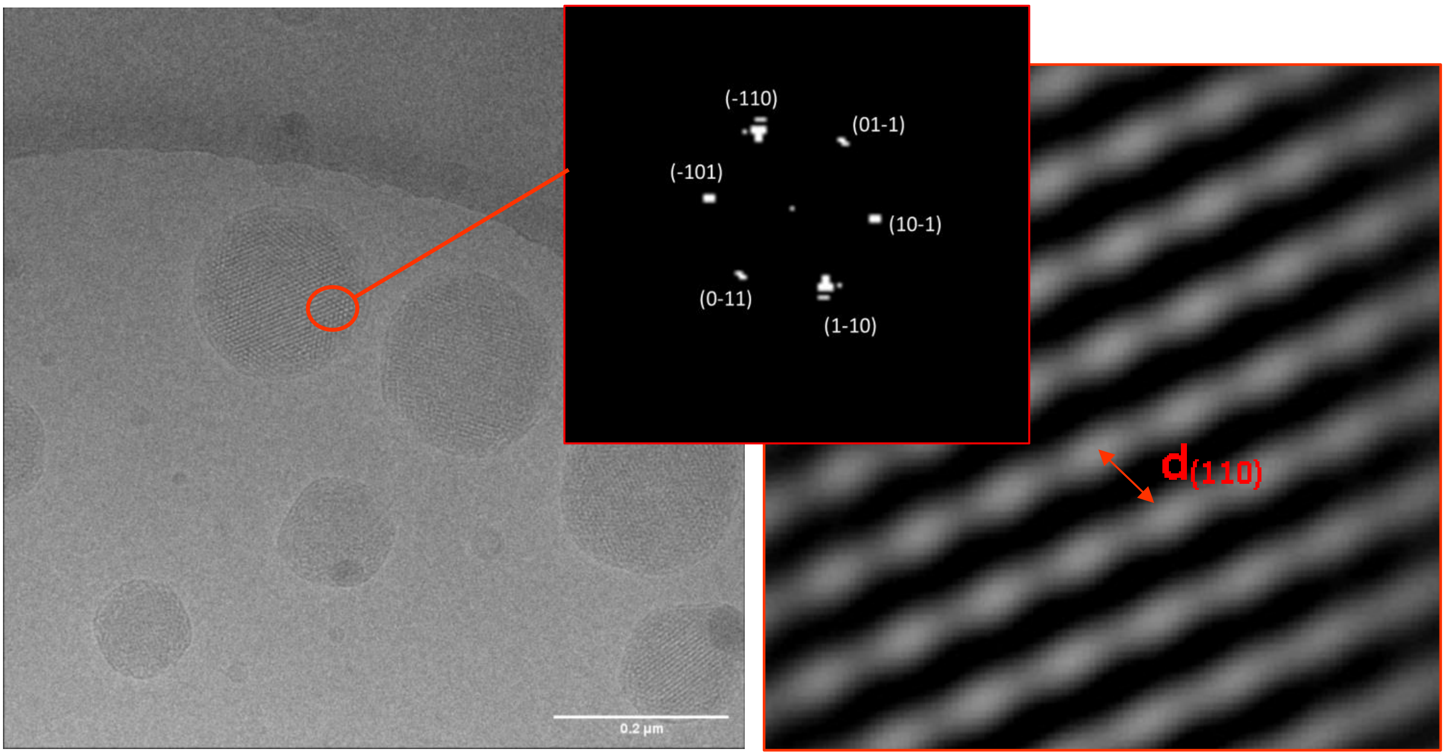

Figure S.7.: A and B: *Supplementary cryo-EM images with insets showing FFT and iFFT of Pn3m cubosomes. A hexagonal pattern of points was observed in the FFT, corresponding to the <111> direction of a Schwarz diamond cubic lattice, onto which miller indices could be assigned. Micrographs also show co-existence of vesicular particles, though to a lesser extent than for monoolein systems. The scale bar indicates 200 nm.*

Phytantriol/Tocopherol Acetate Hexosomes (p6mm) – Cryo - EM

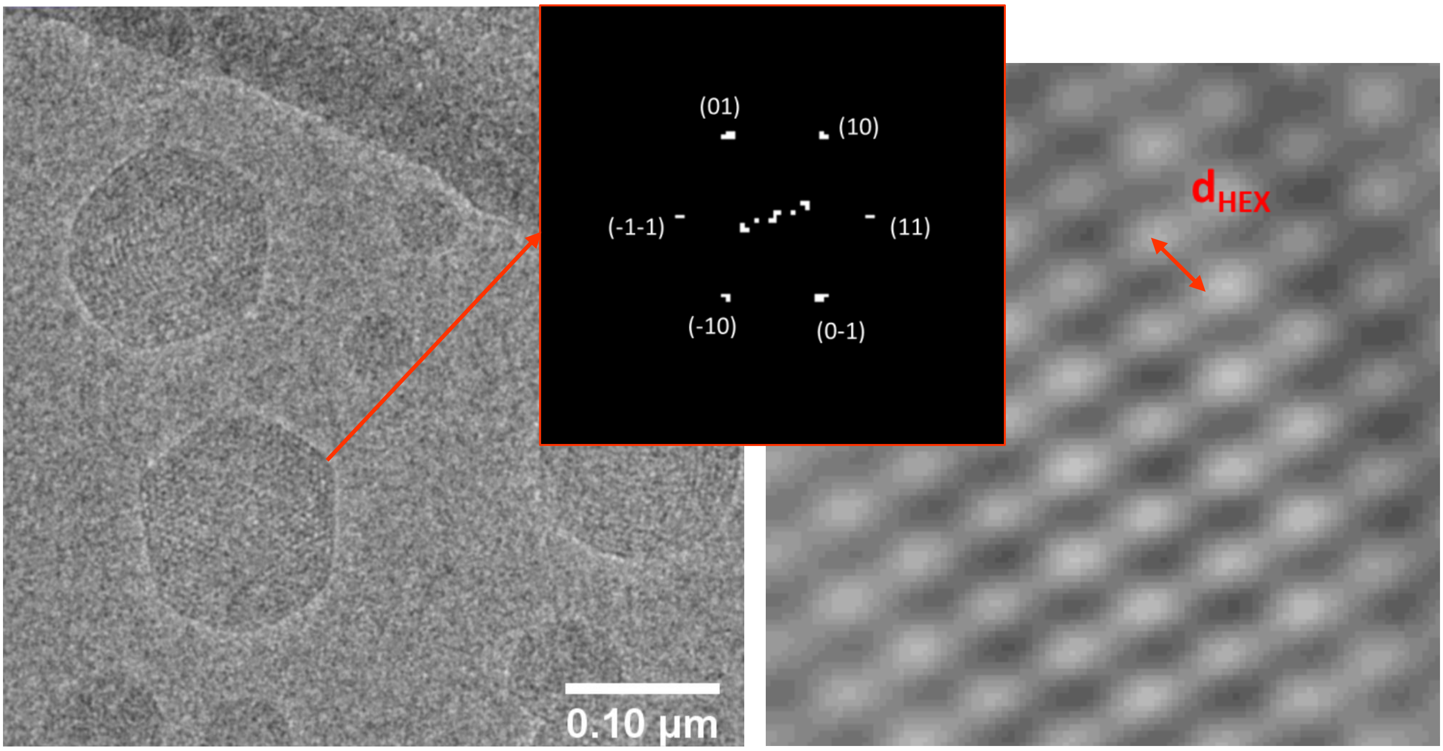

Figure S.8.*: A and B:* *Supplementary cryo-EM images with insets showing FFT and iFFT of p6mm hexosomes. Spinning-top morphopogy is evident. Hexagonal 2-D symmetry noted via FFT, to which miller indices were assigned by matching with a predicted reciprocal lattice. Inverse FFT allowed more accurate determination of* d_{hk}.

Curcumin loading into LLCs – Standard curve

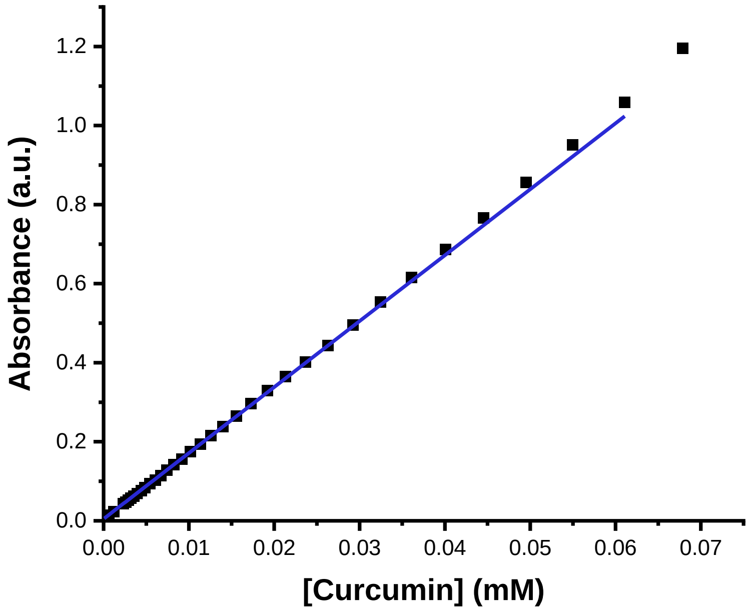

Figure S.9. *Standard curve for curcumin absorbance, measured at 426 nm, against which curcumin pellets were compared after centrifugation.*

Carboxyfluorescein loading into LLCs – Standard curve and assay details

Carboxyfluorescein was loaded into LLCs via microfluidics, as described in the methods and results section of the main article. Samples were columned and assessed for the fluorescence intensity before and after treatment with Triton X-100 (5 wt%) (2.5 μL per 200 μL sample). The chromatograms generated, along with dynamic light scattering measurements, (as shown in SI fig. 5) were used to determine the elution times of each LLC (found to be fraction 6 for each – ie. after 1500 μL dilution buffer had passed through the loaded column). The difference in fluorescence intensity before and after treatment with surfactant was used to determine the release efficiency, following the equation below:

$$\% \, \boldsymbol{release\ efficiency} = \frac{(\boldsymbol{f_f - f_0})}{\boldsymbol{f_f}} \, \boldsymbol{x\ 100}\ \%$$

Where f_f and f_0 are final and initial fluorescence intensity values respectively. It should be noted that this calculation is often used to represent the % loading efficiency of liposomal formulations. This can be misleading however, as concentrations are not considered, and values become artificially large. To calculate % loading efficiency for an end lipid concentration of 1.4 mM, loaded with dye at 50 mM, the value obtained from f_f -f_0 was used to determine the concentration of carboxyfluorescein present in the purified LLC samples by comparing to a standard curve for CBF (shown in SI fig. 5). A dilution factor of approximately 1.6 after purification was taken into account (250 μL crude to 300 μL column fraction).

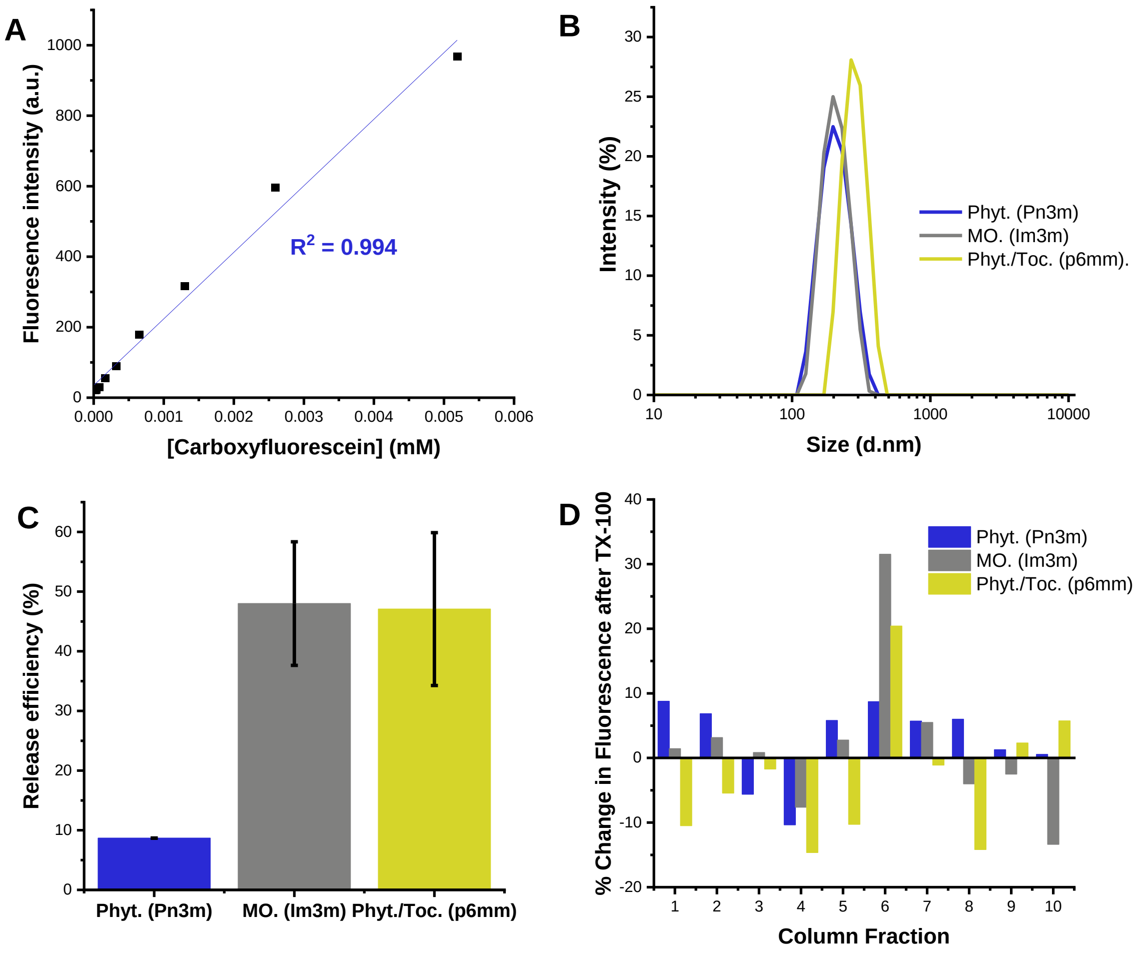

Figure S.10. ***A*** *Standard curve for 5(6) carboxyfluorescein measured at PMT 600 V, with inset showing linear regression equation used for determining CBF concentration present in LLCs after lysis with Triton X-100.* ***B.*** *Dynamic light scattering for LLC particles after column purification (fraction 6), with inset showing corresponding correlograms (mean hydrodynamic size between 200 and 250 nm; PDI values < 0.2.* ***C.*** *Bar chart showing % release efficiency calculated as described in the preceding text, with error bars indicating standard deviation between three separate experiments.* ***D.*** *Release assay data for each LLC after purification. Column fraction 6 (1500 μL elution buffer) was seen to contain LLCs at the highest concentration.*

Effect of cargo type on estimated d-spacing

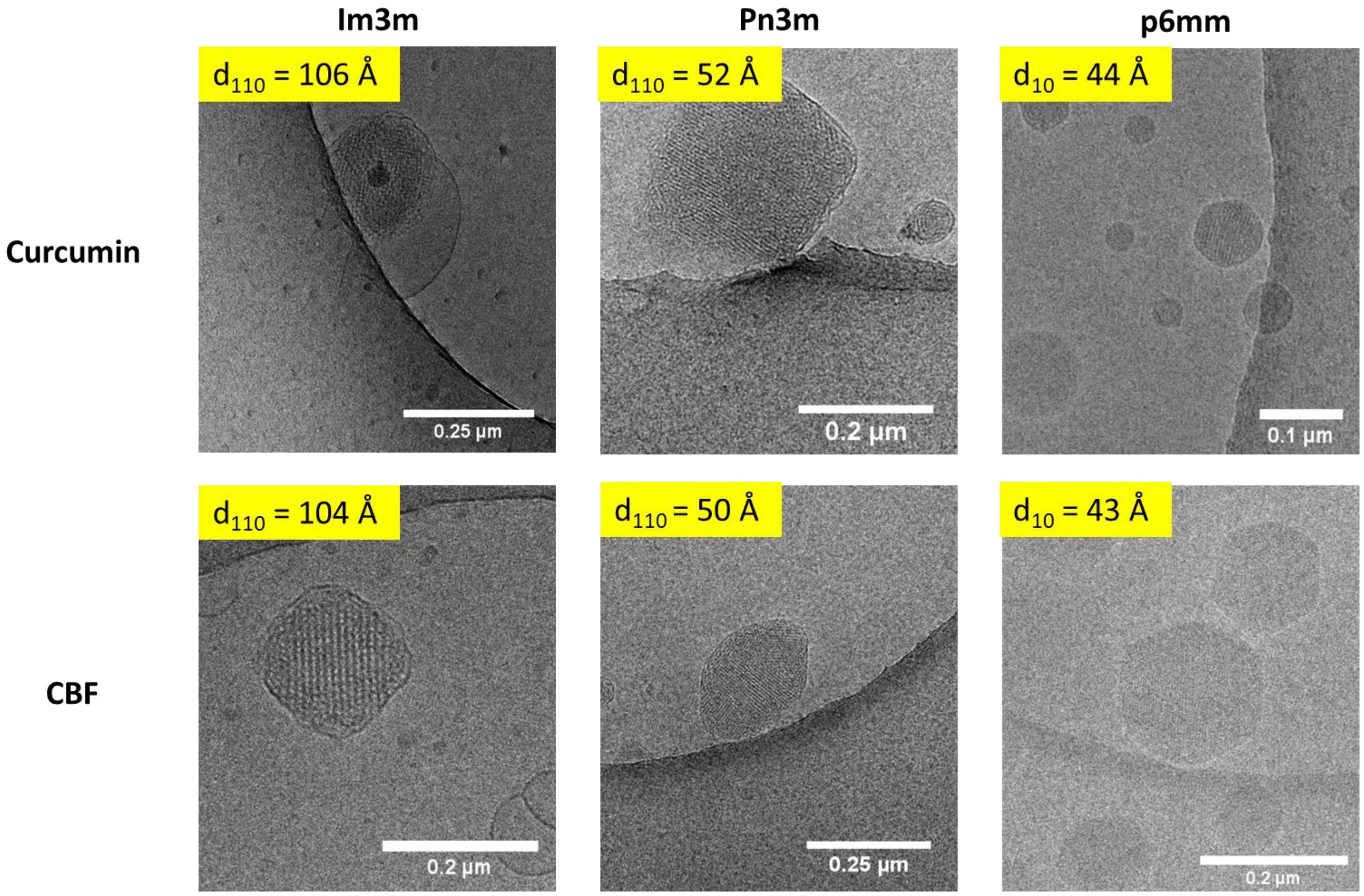

Figure S.11. *Cryo-TEM micrographs for each particle class with Curcumin (top three images) and CBF (bottom three images). Particles were prepared as described in the methods section for cargo encapsulation. Approximate d-spacing values were calculated using brightness-adjusted fast Fourier transforms of an ordered portion of a given particle.*

Calcein delivery to DOPC GUVs – cubosome size dependance

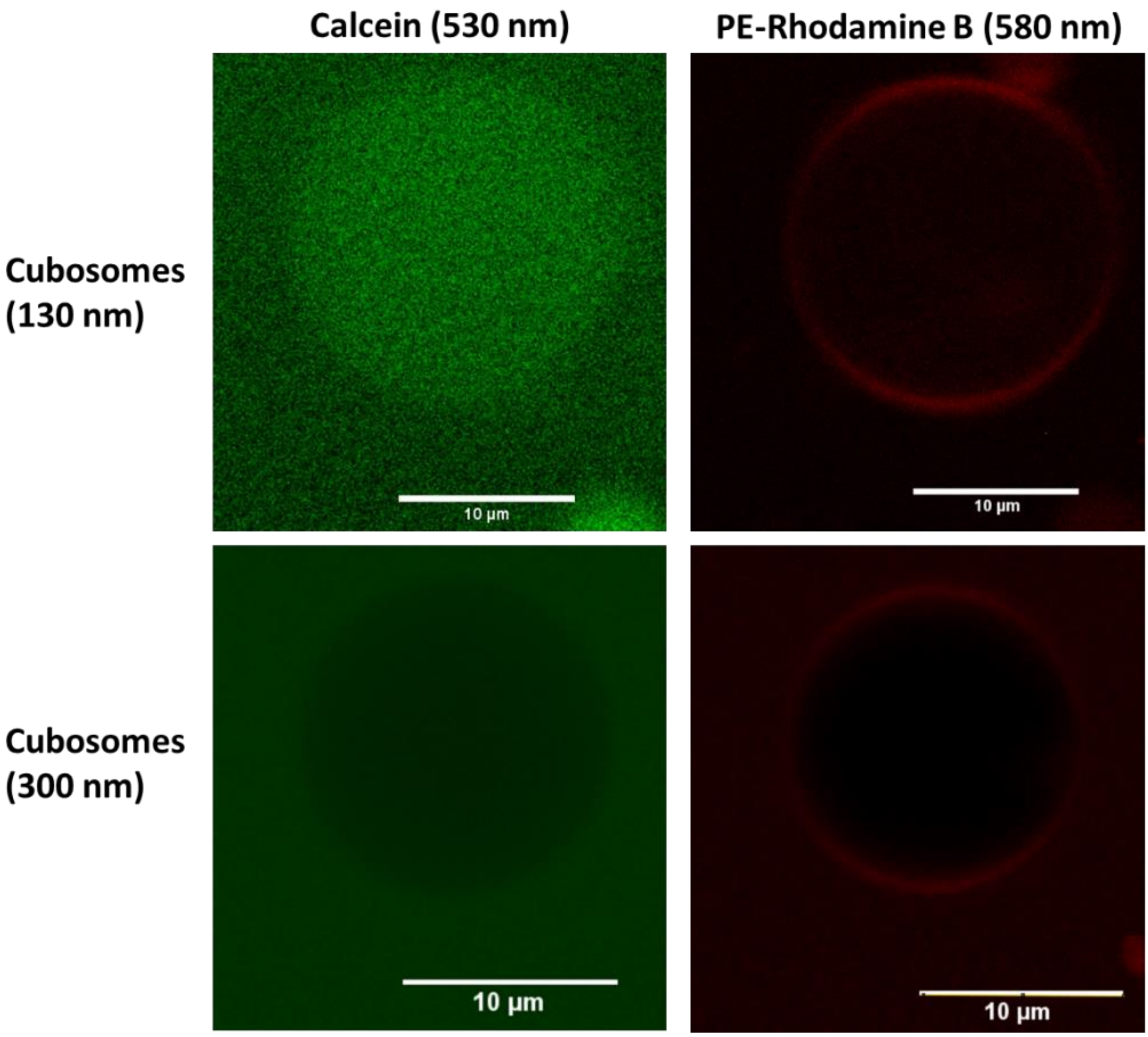

Figure S.12. *Representative images for DOPC GUVs doped with PE Rhodamine B. The top two images shown are the same GUV viewed through the green and red channel respectively, after treatment with calcein loaded cubosomes with 130 nm hydrodynamic diameter. The bottom two images show a GUV after treatment with calcein loaded 300 nm sized cubosomes. Scale bars indicate 10 micron in all images. No obvious disruption of the GUV membrane was observed.*

Tracing cubosome accumulation around DOPC GUV membranes

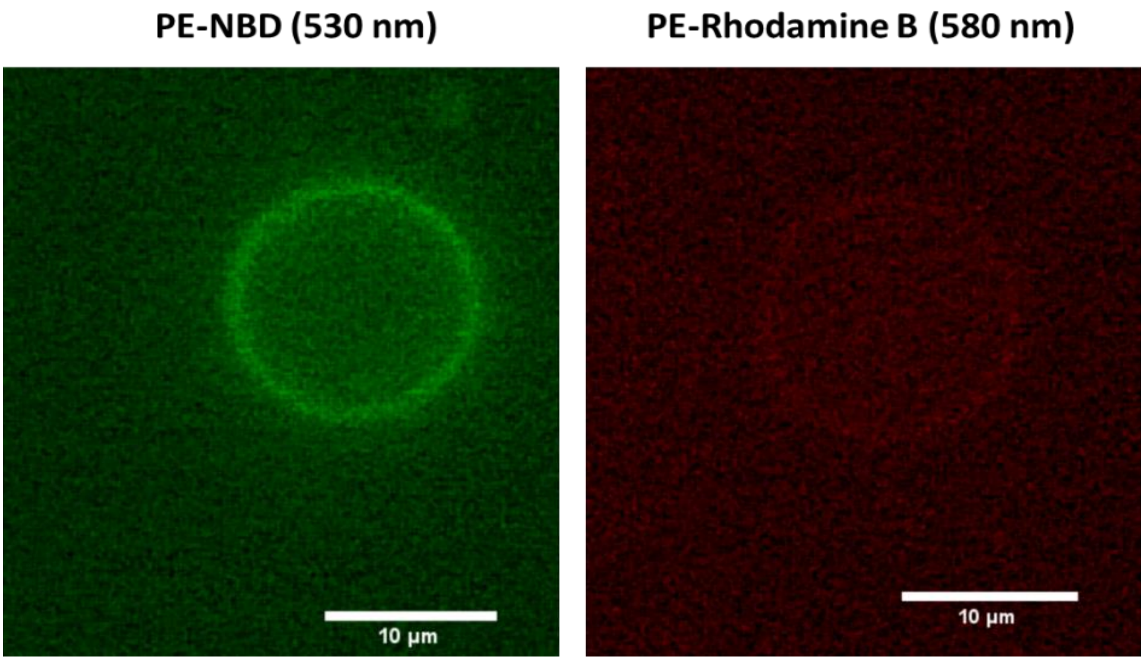

Figure S.13. *Image of DOPC GUV doped with PE-NBD, treated with PE-Rhodamine B labelled cubosomes (130 nm). An accumulation of fluoresence intensity corresponding to Rhodamine B excitation around the GUV membrane was taken to indicate some interaction of cubosome particles with the lamellar membrane of the GUV (either docking, hemi-fusion or full fusion).*
